# Identification of a DNA Damage Response and Repair-Related Gene-Pair Signature for Prognosis Stratification Analysis in Hepatocellular Carcinoma

**DOI:** 10.3389/fphar.2022.857060

**Published:** 2022-04-05

**Authors:** Yi Chen, Mengjia Huang, Junkai Zhu, Li Xu, Wenxuan Cheng, Xiaofan Lu, Fangrong Yan

**Affiliations:** State Key Laboratory of Natural Medicines, Research Center of Biostatistics and Computational Pharmacy, China Pharmaceutical University, Nanjing, China

**Keywords:** DNA damage response and repair, hepatocellular carcinoma, prognosis, HBV, chemotherapy

## Abstract

**Background:** Nowadays, although the cause of hepatocellular carcinoma (HCC) mortality and recurrence remains at a high level, the 5-year survival rate is still very low. The DNA damage response and repair (DDR) pathway may affect HCC patients’ survival by influencing tumor development and therapeutic response. It is necessary to identify a prognostic DDR-related gene signature to predict the outcome of patients.

**Methods:** Level 3 mRNA expression and clinical information were extracted from the TCGA website. The GSE14520 datasets, ICGC-LIRI datasets, and a Chinese HCC cohort were served as validation sets. Univariate Cox regression analysis and LASSO-penalized Cox regression analysis were performed to construct the DDR-related gene pair (DRGP) signature. Kaplan–Meier survival curves and time-dependent receiver operating characteristic (ROC) analysis curves were calculated to determine the predictive ability of this prognostic model. Then, a prognostic nomogram was established to help clinical management. We investigated the difference in biological processes between HRisk and LRisk by conducting several enrichment analyses. The TIDE algorithm and R package “pRRophetic” were applied to estimate the immunotherapeutic and chemotherapeutic response.

**Results:** We constructed the prognostic signature based on 23 DDR-related gene pairs. The patients in the training datasets were divided into HRisk and LRisk groups at median cut-off. The HRisk group had significantly poorer OS than the LRisk group, and the signature was an independent prognostic indicator in HCC. Furthermore, a nomogram of the riskscore combined with TNM stage was constructed and detected by the calibration curve and decision curve. The LRisk group was associated with higher expression of HBV oncoproteins and metabolism pathways, while DDR-relevant pathways and cell cycle process were enriched in the HRisk group. Moreover, patients in the LRisk group may be more beneficial from immunotherapy. We also found that *TP53* gene was more frequently mutated in the HRisk group. As for chemotherapeutic drugs commonly used in HCC, the HRisk group was highly sensitive to 5-fluorouracil, while the LRisk group presented with a significantly higher response to gefitinib and gemcitabine.

**Conclusion:** Overall, we developed a novel DDR-related gene pair signature and nomogram to assist in predicting survival outcomes and clinical treatment of HCC patients. It also helps understand the underlying mechanisms of different DDR patterns in HCC.

## Introduction

Liver cancer remains a major contributor to the global cancer burden, and it is estimated that the global incidence cases will exceed 1 million by 2025 (Llovet et al., 2021). Hepatocellular carcinoma (HCC) is the most common form of liver cancer and the fourth-highest cause of cancer mortality (Villanueva, 2019). Hepatitis B and C virus (HBV and HCV) infection, cirrhosis, metabolic diseases, and alcohol-related liver disease are the main risk factors for HCC (Tunissiolli et al., 2018). Although diagnosis and treatment have made rapid progression in HCC, the 5-year survival rates remain very low (Siegel et al., 2013). Because of the different levels of heterogeneity in HCC, particularly interpatient, intertumor, and intratumor (Hoshida et al., 2009), several prognostic biomarkers widely used in clinical practice are still far from satisfying (Liu et al., 2019). Recently, deep mining of public gene expression data tends to be an effective method to identify novel gene prognostic signatures to accurately predict HCC prognosis and guide personalized therapy for patients (Long et al., 2018; Liu et al., 2019; Liu et al., 2020).

Genomic instability has been reported as a fundamental hallmark of cancer (Negrini et al., 2010). Genomic instability refers to the high frequency of harmful changes in the genomic structure due to DNA damage response (Sahin et al., 2016). To maintain genome stability, eukaryotic cells evolve several mechanisms to detect DNA damage, present damage signals, and mediate cellular responses to eliminate the damage (Ciccia and Elledge, 2010; Pandita et al., 2013; Su et al., 2018). This process is called DNA damage response and repair (DDR). The DDR pathway is an important mechanism that consists of eight major pathways: mismatch repair (MMR), base excision repair (BER), nucleotide excision repair (NER), homologous recombination repair (HRR), checkpoint factors (CPF), nonhomologous end-joining (NHEJ), Fanconi anemia (FA), and translesion DNA synthesis (TLS) (Scarbrough et al., 2016; Song et al., 2021). Furthermore, studies have revealed that the DDR system plays an important role in tumorigenesis, tumor progression, and response to therapy (Lima et al., 2019). It is currently appreciated that tumor progression requires downregulation of DNA damage response mechanisms and an increase in genetic instability to achieve uncontrolled proliferation and adaptability to invasive tumors (Jeggo et al., 2016). For tumor treatment, genotoxic drugs have been the mainstay of cancer chemotherapy for over 30 years, which cause DNA damage exceeding the repair capacity of DDR systems (Pearl et al., 2015).

DDR pathways are found to be associated with chemotherapy resistance of HCC (Evans et al., 2016; Chen Y. et al., 2021). HCC cells strengthen their DDR ability to frustrate the DNA damage caused by chemotherapy drugs, often leading to chemotherapy resistance (Al-Hrout et al., 2018; Chen et al., 2018). Consequently, the DDR pathway may impact HCC patients’ survival by influencing tumor development and therapeutic response. Recently, some studies have successfully constructed prognostic and predictive signatures based on the expression of the DDR gene (Evans et al., 2016; Sharma et al., 2019; Chen J. et al., 2021). Taken together, it is significant to explore a prognostic DDR-related gene signature to predict the outcome and characterize two different DDR pathway activity subtypes of HCC patients.

In this study, a gene-pair strategy was used to improve the robustness of the identification of the predictive signature (Eddy et al., 2010; Li et al., 2017). Univariate and Lasso-Cox regression analysis was conducted to construct a novel prognostic biomarker. We clustered HCC patients into two risk groups according to 23 DDR-related gene pairs and identified two subtypes related to prognosis and chemotherapy response. In addition, the prognostic value of our DDR-related gene pair signature was further validated in GSE14520 datasets, ICGC-LIRI datasets, and a Chinese HCC cohort (LIHC-CN). Collectively, we identified a robust signature to present new evidence into the prognostic value of the expression of DDR-related genes in HCC and explore the underlying mechanisms of DDR patterns and potential therapeutic drugs in HCC treatment.

## Materials and Methods

### Data Collection and Processing

Level 3 mRNA expression, somatic mutation data, and clinicopathological data were obtained from the TCGA website (https://portal.gdc.cancer.gov/repository). A segment of copy number for the TCGA-LIHC cohort was accessed from the GDAC FireBrowse (http://firebrowse.org/). The raw count data were transferred to transcripts per kilobase of exon model per million mapped reads (TPM) data which would represent the expression of mRNA in the TCGA-LIHC cohort. After filtering mRNAs with low median absolute deviation (mad ≤0.5) across all samples and removing the samples without complete survival information, a total of 351 HCC samples were enrolled in this study. RNA-seq data, somatic mutation data, and clinical data with 240 tumor samples were downloaded from the International Cancer Genome Consortium (ICGC) portal (https://dcc.icgc.org/projects/LIRI-JP). Raw read count values were transformed into TPM values for subsequent analysis. The expression data and detailed clinical information of GSE14520 (including 219 HCC samples based on the GPL3921 platform) were downloaded from the Gene Expression Omnibus (GEO) (http://www.ncbi.nlm.nih.gov/geo/). Additionally, a LICH-CN cohort with 159 Chinese HCC patients was downloaded for somatic mutation, clinical outcome, and transcriptome expression FPKM value from the literature (Gao et al., 2019).

### Construction and Validation of the DDR-Related Gene Pair Signature

DDR-gene list including 557 genes was assembled from relevant gene lists, including MSigDB from the Broad Institute (http://www.broad.mit.edu/gsea/msigdb/) or literature (Pearl et al., 2015; Knijnenburg et al., 2018; Chen J. et al., 2021). Finally, 384 DDR genes detected in all datasets were analyzed in this study (Supplementary Table S1). Then, each gene pair was calculated *via* their gene expression level in each HCC sample. According to the pairwise comparison, the calculated score was 0 when the first expression level of the DDR gene was higher than that of the following DDR gene; otherwise, the calculated score was 1. DRGP scoring 0 or 1 in more than 90% of the samples were removed because they could not provide discriminative patients with different survival. The remaining DRGPs were considered as initial candidate DRGPs.

Patients were randomly divided into training and testing sets at cut-of 7:3 in the TCGC cohort. Then, a univariate Cox regression analysis was performed to identify the significant DRGPs related to overall survival (OS) if the FDR *p*-value was less than 0.05. Next, candidate DRGPs were submitted to LASSO-penalized Cox regression analysis based on package “glmnet” in R to construct an optimal prognostic signature in TCGA training datasets (Friedman et al., 2010). A DDR-related gene pair riskscore of each sample was calculated based on the lasso Cox regression model coefficients (β) multiplied with its DRGP score, as follows:

$$Riskscore=\sum_{i=1}^{n}\left(\beta_{i}\times Score_{i}\right)$$

where *Score*_*i*_ is the relative expression of DRGPs for patient *j* in each cohort and *β*_*i*_ is the LASSO Cox coefficient of the DRGPs_*i*_. Then, all patients were separated into low- (LRisk) or high-risk (HRisk) groups at the median cut-off. Kaplan–Meier survival curves were plotted for prediction of the clinical outcomes in the two groups *via* the “survival” package in R. The differences in survival were evaluated *via* the log-rank test. Time-dependent receiver operating characteristic (ROC) analysis curves were built, and the area under the curves (AUCs) for 1-, 3-, and 5-year overall survival (OS) were calculated utilizing the “survivalROC” package in R (Heagerty et al., 2000). The same method was further investigated in the TCGA testing cohort, TCGA whole cohort, GSE14520 cohort, and ICGC-LIRI cohort.

### Subgroup Kaplan-Meier Survival Analysis

To explore the diagnostic capability of the DRGP prognostic signature in different levels of other clinical prognostic parameters, HCC samples in TCGA sets were stratified into different subgroups based on age (≥60 and <60), gender (female and male), TNM stage (I and II + III + IV), grade (G1+G2 and G3+G4), and *TP53* (mutation and wild). Then, cancer samples in each subgroup were clustered into HRisk and LRisk groups. The differences in prognosis between the two groups were assessed *via* Kaplan–Meier OS analysis, followed by a log-rank test.

### Correlations Between the DRGP Model and Clinical Properties

To elucidate whether the prognostic model for OS is independent of other prognostic factors, we presented univariate Cox regression analysis and multivariate Cox regression survival analysis to predict the clinical outcomes of HCC patients, which was visualized *via* package “forestplot” in R. Hazard ratio (HR), 95% confidence interval (CI), and *p*-value were calculated, respectively.

### Construction and Validation of Gene Prognostic Nomogram

A nomogram was constructed based on all independent prognostic parameters screened by univariate and multivariate Cox proportional hazards regression analysis to predict the probability of 1-, 3-, and 5-year OS using the “rms” package of R software. Then, we used a calibration curve to visualize the performance of the nomogram with the observed rates of the TCGA whole set at corresponding time points by a bootstrap method with 1000 resamples. Furthermore, decision curve analysis (DCA) and calibration curves were detected to check the reliability of our nomogram (Kerr et al., 2016).

### Functional Enrichment Analysis

To investigate the difference in biological process between HRisk and LRisk, we performed some enrichment analysis using “GSVA” and “clusterprofiler” R packages (Yu et al., 2012; Hänzelmann et al., 2013). The infiltrating score of 24 microenvironment cell types was calculated with single-sample gene set enrichment analysis (ssGSEA) in the “GSVA” R package. Gene set enrichment analysis (GSEA) was conducted between HRisk and LRisk by using the R “clusterProfiler” package. A signature of eleven oncogenic pathways and a DDR gene list, which include eight core DDR pathways, were obtained from the literature (Pearl et al., 2015; Sanchez-Vega et al., 2018; Lu et al., 2021). Then, we used the gene set variation analysis (GSVA) method to generate enrichment scores for each cohort using the R package “GSVA”. The KEGG gene sets (c2.cp.kegg.v7.4.symbols.gmt) was selected as the reference datasets, which was obtained from the MSigDB database.

### Prediction of Immunotherapeutic and Chemotherapeutic Response

For immunotherapy, the tumor immune dysfunction and exclusion (TIDE) algorithm (http://tide.dfci.harvard.edu/) was applied to predict potential clinical response to immune checkpoint inhibitors (Jiang et al., 2018). Based on Genomics of Drug Sensitivity 2016 (GDSC 2016; https://www.cancerrxgene.org/), the R package “pRRophetic” was applied to estimate the chemotherapeutic sensitivity by the half-maximal inhibitory concentration (IC_50_) of each HCC sample in four cohorts. Therefore, we could investigate the different sensitivity of common liver cancer chemotherapy drugs between HRisk and LRisk. In addition, in order to identify potential drugs in HCC samples, we performed a two-step analysis to find candidate compounds as described previously (Yang et al., 2021). First, differential drug response analysis between top decile riskscore samples and bottom decile riskscore samples was conducted to verify drugs with significantly different estimated IC_50_ in two riskgroups (|log2FC| > 0.2). Next, Spearman correlation analysis was utilized to calculate the correlation coefficients between riskscore and IC_50_ of each candidate drug (|Spearman correlation coefficient| > 0.4).

### *HBV*_*pca*_ Quantifies the Expression Level of HBV Virus

HBV oncoproteins were quantified for expression according to the previous study: HBVgp2_S, HBVgp3_X, HBVgp4_c, and HBVgp2_pre-S1/S2 (Xue et al., 2021). The four HBV oncoprotein expressions were identified and presented as FPKM values. To comprehensively explain the original expression level of HBV oncoproteins, we established a variable that was calculated by principal component analysis (PCA) as the previous study described (Lu et al., 2019). HBV_*pca*_ was derived from the first and second principal components that represented 76.73 and 17.10% of the variation in the original data, respectively. The coefficients of four HBV oncoproteins to the first and second principal components are shown in Table 1.

**TABLE 1:** Coefficients of four HBV oncoproteins to the first and second principal components.

Principal components	HBV oncoproteins			
	HBVgp2_S	HBVgp3_X	HBVgp4_c	HBVgp2_pre-S1/S2
Componment1	0.62	0.57	0.34	0.41
Componment2	0.29	0.43	−0.74	−0.42

Mathematically, let *E*_*ij*_ denotes the log_2_(FPKM +1) value of specific oncoprotein *j* in sample *i*, and C_*jk*_ represents the corresponding coefficient of HBV oncoprotein (*HBV*_*j*_; *j*∈{1,2,3,4}) for principal component *k* (*k*∈{1,2}). The *HBV*_*pca*_ can be calculated as follows:

$$HBV_{pca}=\;\left[\begin{array}{ccc} E_{11} & \cdots & E_{1j}\\ \vdots & \ddots & \vdots \\ E_{i1} & \cdots & E_{ij} \end{array}\right]\left[\begin{array}{ccc} C_{11} & \cdots & E_{1k}\\ \vdots & \ddots & \vdots \\ C_{j1} & \cdots & E_{jk} \end{array}\right]\left[\begin{array}{c} 0.7673\\ 0.1710 \end{array}\right]$$

### Comprehensive Analysis of Genomic Variation Between Different DRGP Subgroups

We then investigated the genomic variation between HRisk and LRisk groups. The mutation landscape was analyzed by the R package “maftools” with the initial removal of 100 FLAGS (Mayakonda et al., 2018). The data CNV segments were detected by Genomic Identification of Significant Targets In Cancer 2.0 (GISTIC 2.0) analysis. In the process of GISTIC 2.0 analysis, except for the refgene file which was “Human_Hg19.mat”, parameters were set to the default parameters. The individual fraction of genome altered (FGA), fraction of genome lost (FGL) and fraction of genome gained (FGG) for the HCC in the TCGA cohort were calculated as the study described (Lu et al., 2021). We also obtained GISTIC calls comprising −2 (deletion), −1 (loss), 0 (diploid), 1 (gain), and 2 (amplification) from GISTIC2.0 (Wu et al., 2020).

### Statistical Analyses

All statistical analyses were performed with R software (version 4.1.1: http://www.r-project.org) and R Bioconductor packages in this study. Kaplan–Meier analysis with the log-rank test was used to detect differences of OS between different groups through the package “survminer” in R. Time-dependent ROC was utilized to evaluate the predictive accuracy of the DRGP riskscore through package “survivalROC” in R. Cox proportional hazards regression for estimating the hazard ratios (HRs) and 95% confidence interval (CI). Comparison of a continuous variable in two groups was performed using Wilcoxon rank-sum test. Correlation between two continuous variables was measured by Spearman’s rank-order correlation. Differences in proportions were compared by the Chi-squared test or Fisher’s exact test.

## Results

### Construction and Validation of the Prognostic DDR-Related Gene Pair Signature

**FIGURE 1:**
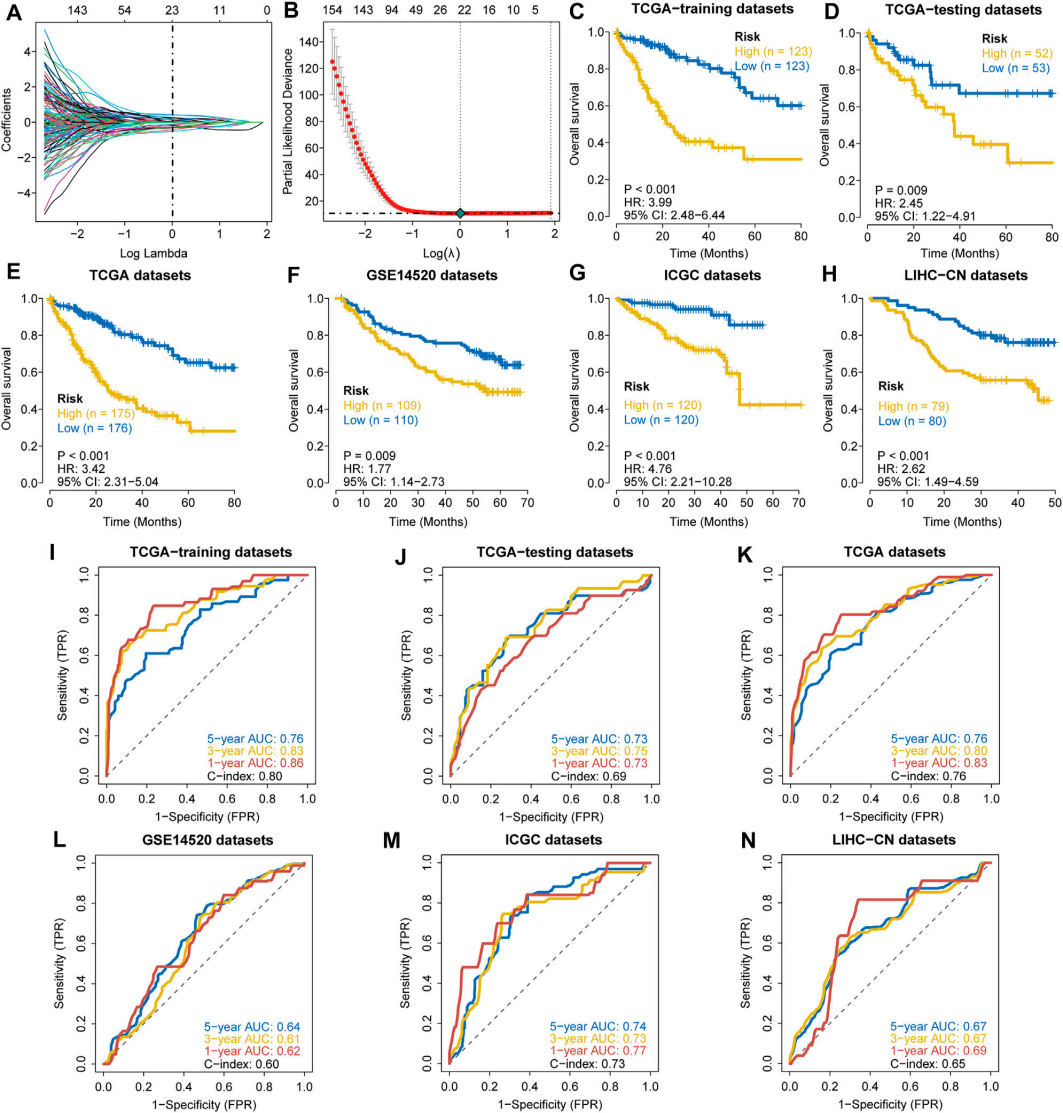
Construction and validation of a prognostic DDR-related gene pair signature. **(A,B)** LASSO regression identified 23 DRGPs. **(C–H)** The Kaplan–Meier overall survival (OS) curves in the TCGA training datasets **(C)**, TCGA testing datasets **(D)**, TCGA datasets **(E)**, GSE14520 datasets **(F)**, ICGC datasets **(G)**, and LIHC-CN datasets **(H)** show that patients in the HRisk group have a poorer prognosis. **(I–N)** ROC curves show the predictive efficiency of the signature for patients in the TCGA training datasets **(I)**, TCGA testing datasets **(J)**, TCGA datasets **(K)**, GSE14520 datasets **(L)**, ICGC datasets **(M)**, and LIHC-CN datasets **(N)** on the survival rate.

The clinical features of HCC samples in the training and validation sets are listed in Supplementary Table S2. In the training datasets of 246 patients, 85 patients died during the follow-up. As tested by the univariate Cox regression OS analysis, 459 DRGPs had significant associations with OS of HCC in the training set (all FDR *p*-value < 0.05). Based on LASSO-Cox regression analysis, 23 DRGPs were independently related to the prognosis of HCC (Figures 1A,B). Among them, 11 gene pairs were risk factors for HCC prognosis (HR > 1). Supplementary Table S3 lists the 23 selected gene pairs and their coefficients. The regression coefficients and DRGP score of these 23 gene pairs in each sample were used to calculate the riskscore in each HCC cohort.

The patients in the training datasets were divided into HRisk or LRisk groups at median cut-off. Kaplan-Meier survival analysis indicated that the HRisk group have a poorer OS than the LRisk group (hazard ratio (HR) = 3.99, 95% CI = 2.48–6.44, *p*-value < 0.001, Figure 1C). The values of AUC are 0.76, 0.83, and 0.86 at 1-, 3-, and 5-year follow-up, respectively (Figure 1I), showing that the signature displays good sensitivity and specificity. The C-index of the DRGP model is 0.80. To determine the predictive ability of this prognostic model, we calculated individual riskscore with the aforementioned method and classified the patients in TCGA-testing set, TCGA whole set and other validation sets into HRisk and LRisk groups. Similarly, we validated the prediction of signature in these datasets. Consistent with the above findings, the HRisk patients in all cohorts have a markedly shorter OS than those in the LRisk group (Figures 1D–H). The AUCs of ROC curves for 1-,3-, and 5- year OS are shown in Figures 1J–N.

### Independent Prognostic Role of the DRGP Signature

**FIGURE 2:**
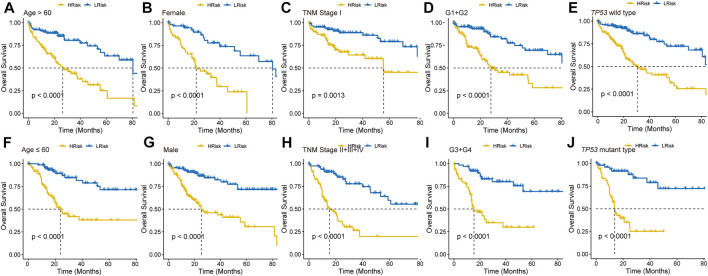
Kaplan–Meier curves analyses of different clinical subgroups in TCGA cohort. Patients were classified into **(A)** Age >60 years, **(B)** Gender: Female, **(C)** TNM stage: I, **(D)** Grade: G1+G2, **(E)** *TP53* wild type, **(F)** Age ≤60 years, **(G)** Gender: Male, **(H)** TNM stage: II + III + IV, **(I)** Grade: G3+G4, and **(J)** *TP53* mutant type.

**FIGURE 3:**
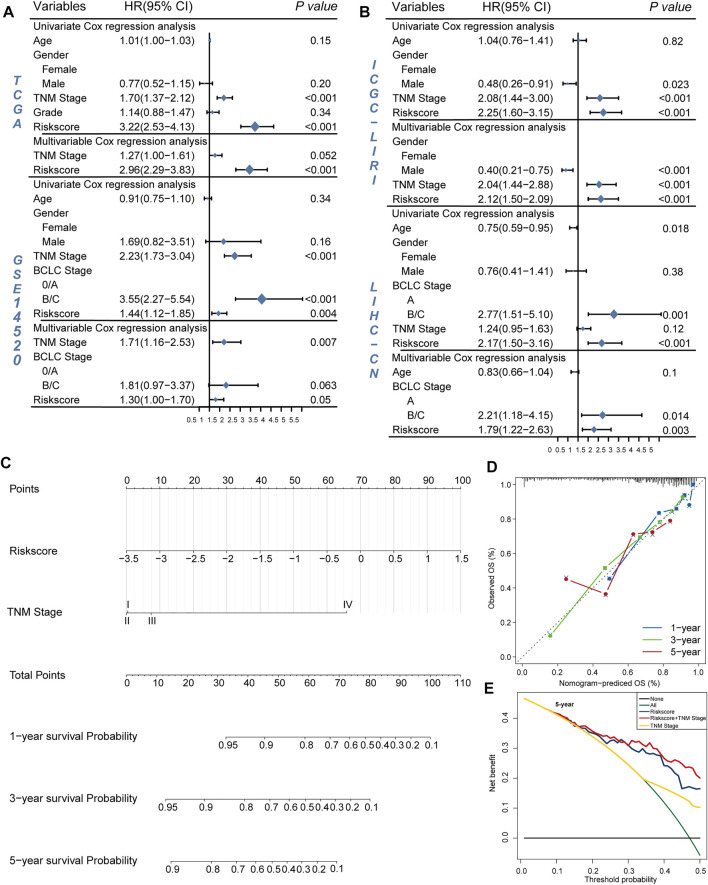
Validation of the independency of the riskscore for prediction of HCC prognosis. **(A,B)** Univariate and multivariate cox regression survival analysis validated riskscore was an independent prognosis factor for HCC patients in TCGA datasets **(A)**, GSE14520 datasets **(A)**, ICGC datasets **(B)**, LIHC-CN datasets **(B)**. The *p-*value, hazard ratio (HR), and 95% confidence interval (CI) were indicated in the forest plots. The blue circle represents the value of HR each parameter scored. **(C,E)** Construct nomogram for survival prediction. **(C)** which integrated with two independent prognosis factors for predicting the probability of patient mortality at 1-, 3-, or 5-year OS. **(D)** The calibration plots for predicting patient 1-, 3-, or 5-year OS. **(E)** DCA curves for two independent prognostic factors or a combination of them in OS prediction.

To further explore the clinical potentiality of the prognosis model in HCC, stratified analysis based on these clinical characteristics was conducted. As shown in Figures 2A–J and Supplementary Figure S1, Kaplan–Meier OS curves also showed that HRisk patients had considerably worse OS than LRisk patients, which further indicated the excellent prediction of the DRGP model. We further analyzed whether the riskscore was an independent prognostic predictor for OS. In univariate Cox regression analyses, high riskscore was significantly associated with shorter OS in TCGA cohort (HR = 3.22, 95% CI = 2.53–4.13, *p-*value < 0.001, Figure 3A) According to the multivariate Cox regression analysis results, we considered the TNM stage (HR = 1.27, 95% CI = 1.00–1.61, *p*-value = 0.0515) and riskscore (HR = 2.96, 95% CI = 2.29–3.83, *p*-value < 0.001] are both independent prognosis factors for TCGA (Figure 3A). The independence of the DRGP signature for HCC prognosis was also confirmed in GSE14520, ICGC, and LIHC-CN cohorts (Figures 3A,B). Collectively, the signature was an independent prognostic factor for HCC.

### Construction and Verification of a Prognostic Prediction Nomogram for HCC

We constructed a nomogram based on multivariate Cox regression analysis for prediction of the 1-, 3-, and 5-year survival probability in TCGA datasets (Figure 3C). As shown in the calibration chart (Figure 3D), the nomogram could robustly predict OS for HCC patients. Moreover, the DCA curve suggested that riskscore was more beneficial when compared with the TNM stage alone (Figure 3E). The DCA curve demonstrated that the net benefit of the combined model was comparable to the riskscore. These results showed that the nomogram built with the combined model might help clinical management.

### LRisk Group Associated With Higher HBV Virus Expression and Higher Proportion of TIDE-Predicted Responders

**FIGURE 4:**
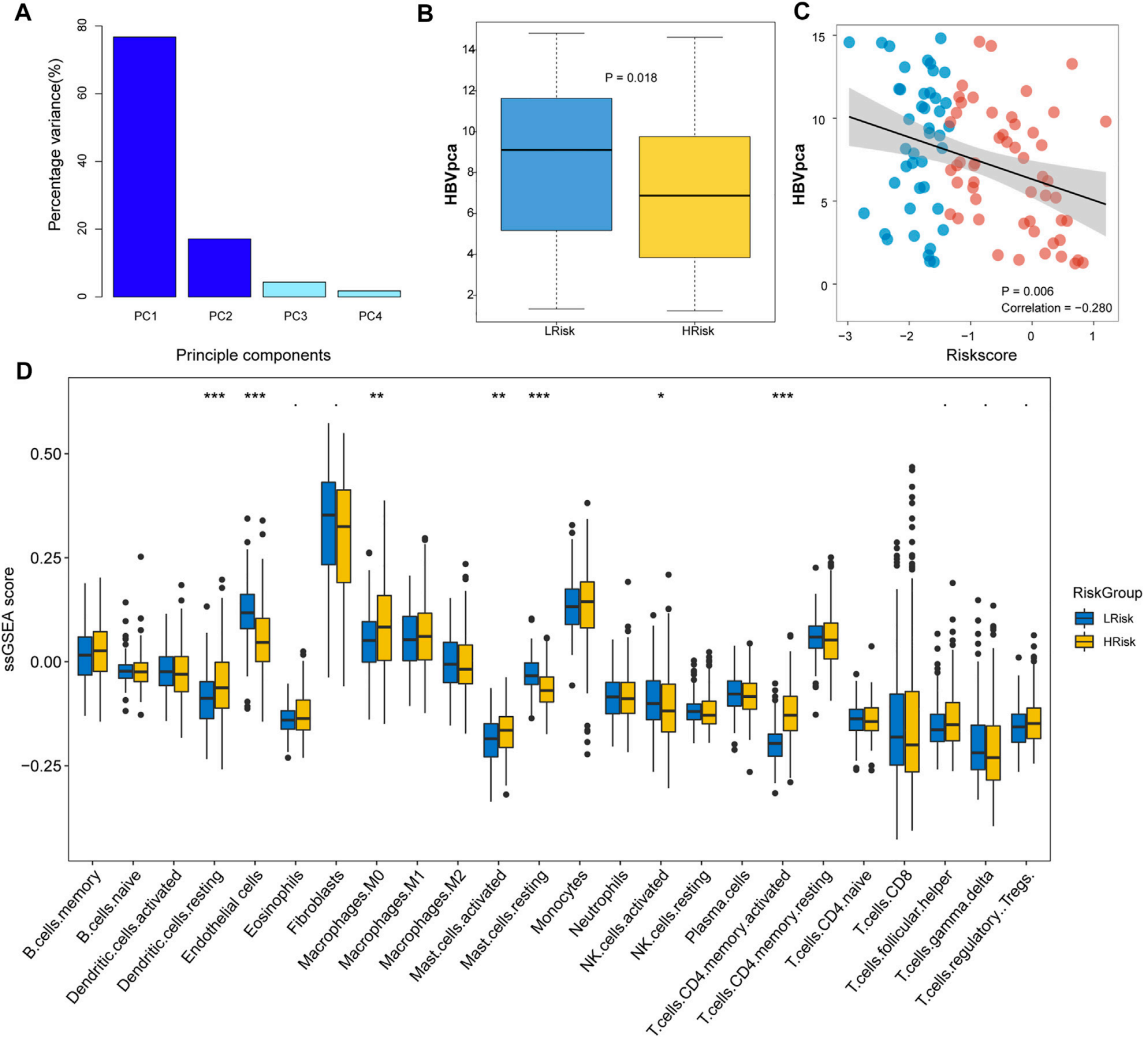
Immune infiltration and *HBV*_*pca*_ score between HRisk and LRisk group in TCGA cohort. **(A)** Barplots showing that the first and second principal components present almost all the variations with a summing percentage of 93.83%. **(B)** Boxplot showing *HBV*_*pca*_ difference in the HRisk and LRisk groups (Wilcoxon test, *p* = 0.018). **(C)** Spearman correlation analysis between *HBV*_*pca*_ and riskscore (*R* = -0.280, *p* = 0.006). **(D)** Boxplot for TCGA cohort showing the enrichment level of 24 microenvironment cell types between HRisk (yellow) and LRisk (blue) groups. Statistical *p* values were calculated by the Wilcoxon test and represented by. < 0.1, * <0.05, ** <0.01, and *** <0.001.

A previous study indicated that the high expression of HBV16 E6/E7 was significantly linked to a favorable prognosis because the inflammatory/immune response of the host may be stimulated (Lu et al., 2019). To investigate whether HBV oncoproteins were differentially expressed between two risk groups in HCC, we calculated the *HBV*_*pca*_ by principal component analysis (PCA) based on the FPKM value of four HBV oncoproteins in the TCGA cohort. In our study, 96 HVB-infected HCC patients were identified, the first and second principal components were used since they covered almost the variations (93.83%; Figure 4A). We found a significant difference in HBV virus expression between HRisk and LRisk (Wilcoxon test, *p* = 0.018, Figure 4B), and *HBV*_*pca*_ has also observed a mildly negative correlation with riskscore (*R* = −0.280, *p* = 0.006, Figure 4C). It means a high level of *HBV*_*pca*_ corresponds to low risk in HCC patients.

To evaluate the tumor immune microenvironment in different groups, a ssGSEA method was used to estimate the infiltration levels of the 24 types of immune cells. The ssGSEA score and immune cell types which were differentially infiltrated between LRisk and HRisk groups in the TCGA set are presented in Figure 4D. The proportion of 24 immune cells in each group is shown in a bar plot. The results revealed that the level of infiltration of dendritic.cells.resting, macrophages.M0, mast.cells.activated, and T.cells.CD4.memory.activated in the HRisk group was significantly higher than that in the LRisk group, while the level of endothelial cells, mast.cells.resting, and NK cells.activated in the LRisk were higher than that in the HRisk group. For further investigating the immune landscape of different risk groups reflected by the DRGP signature, validation cohorts GSE14520, ICGC, and LIHC-CN were also calculated by ssGSEA to verify the differences in risk groups at the immune level (Supplementary Figure S2).

**FIGURE 5:**
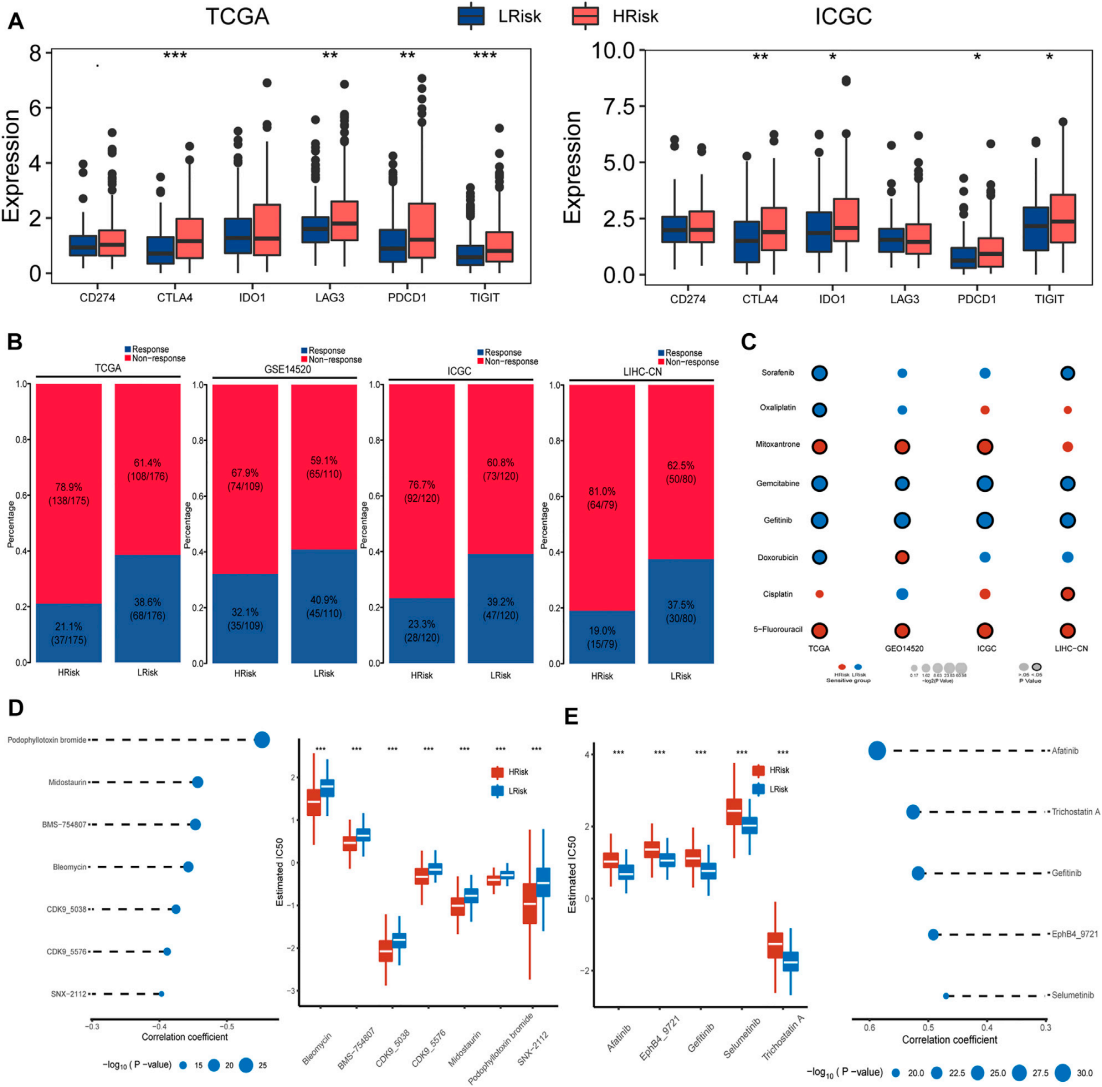
Differential sensitivity to immunotherapy and chemotherapies between HRisk and LRisk. **(A)** Boxplot for TCGA and ICGC cohort showing the different expression levels of six immune checkpoint genes between riskgroups. Statistical *p* values were calculated by the Wilcoxon test and represented by. < 0.1, * <0.05, ** <0.01, and *** <0.001. **(B)** Barplots revealed that LRisk might be more likely to response to immunotherapy than HRisk in TCGA (Chi-square test, *p* < 0.001), GSE14520 (Chi-square test, *p* = 0.23), ICGC (Chi-square test, *p* = 0.012), and LIHC-CN (Chi-square test, *p* = 0.016) cohorts, respectively. **(C)** Bubble plot showing the drug sensitivity for eight commonly used chemotherapeutic drugs in liver cancer between HRisk and LRisk across four cohorts, where red and blue bubbles show that either HRisk or LRisk group is more sensitive to a drug according to the corresponding mean value of predicted IC_50_; and a black circle wrapped around the bubble presents whether a statistically significant difference is achieved. Statistical *p* values were calculated by the Wilcoxon test. **(D)** The results of Spearman’s correlation analysis and boxplots for the distribution of seven HRisk sensitivity drugs response analyses in TCGA datasets. **(E)** The results of Spearman’s correlation analysis and boxplots for the distribution of five LRisk sensitivity drugs response analyses in TCGA datasets.

We also detected and compared the expression levels of several immune checkpoints between the LRisk and HRisk groups. Results showed that the mRNA expression levels of CTLA4, PDCD1, and TIGIT were consistently overexpressed in the HRisk in TCGA and ICGC datasets (Figure 5A), but we could not observe a significant difference in GSE14520 and LIHC-CN cohorts (both, *p* > 0.05, not shown). These results suggested that the HRisk group may contribute to tumor immune dysfunction and immune exclusion in HCC. To investigate whether LRisk responds to immune checkpoint inhibits, we harnessed the TIDE algorithm to predict the potential response to immunotherapy in different groups. The higher TIDE score represented less promising treatment for response to immunotherapy. In our results, the HRisk group contained lower proportion of TIDE-predicted responders than LRisk group in all four cohorts (TCGA (*p* < 0.001), GSE14520 (*p* = 0.23), ICGC (*p* = 0.012), and LIHC-CN (*p* = 0.016); Figure 5B). These results suggest that HCC patients in LRisk might be more beneficial from immune checkpoint inhibitors.

### Prediction the Sensitivity and Chemotherapy

Considering that chemotherapy is a common way to treat liver cancer, we first verify whether the DDR patterns groups may affect the sensitivity of chemotherapeutic drugs commonly used for treating liver cancer (including cisplatin, 5-fluorouracil, gemcitabine, oxaliplatin, doxorubicin, mitoxantrone, gefitinib, and sorafenib). We found that HRisk groups of all four HCC cohorts were highly sensitive to 5-fluorouracil (all, *p* < 0.05; Figure 5C, Supplementary Figure S3), and four LRisk groups presented with a significantly higher response to gefitinib and gemcitabine (all, *p* < 0.05; Figure 5C, Supplementary Figure S3). Next, we performed a two-step analysis to find potential therapeutic compounds. Eventually, the analysis obtained seven compounds (including CDK9_5576, CDK9_5038, bleomycin, midostaurin, SNX-2112, BMS-754807, and podophyllotoxin bromide) that had lower IC_50_ in HRisk and a negative correlation with riskscore (Figure 5D, Supplementary Figure S4) across four datasets and five compounds (including trichostatin A, gefitinib, afatinib, selumetinib, and EphB4_9721) were observed to present a significant response to LRisk and a positive correlation with riskscore (Figure 5E, Supplementary Figure S4).

### Characterization of the HCC Riskgroups Regarding Different Functional Pathways

**FIGURE 6:**
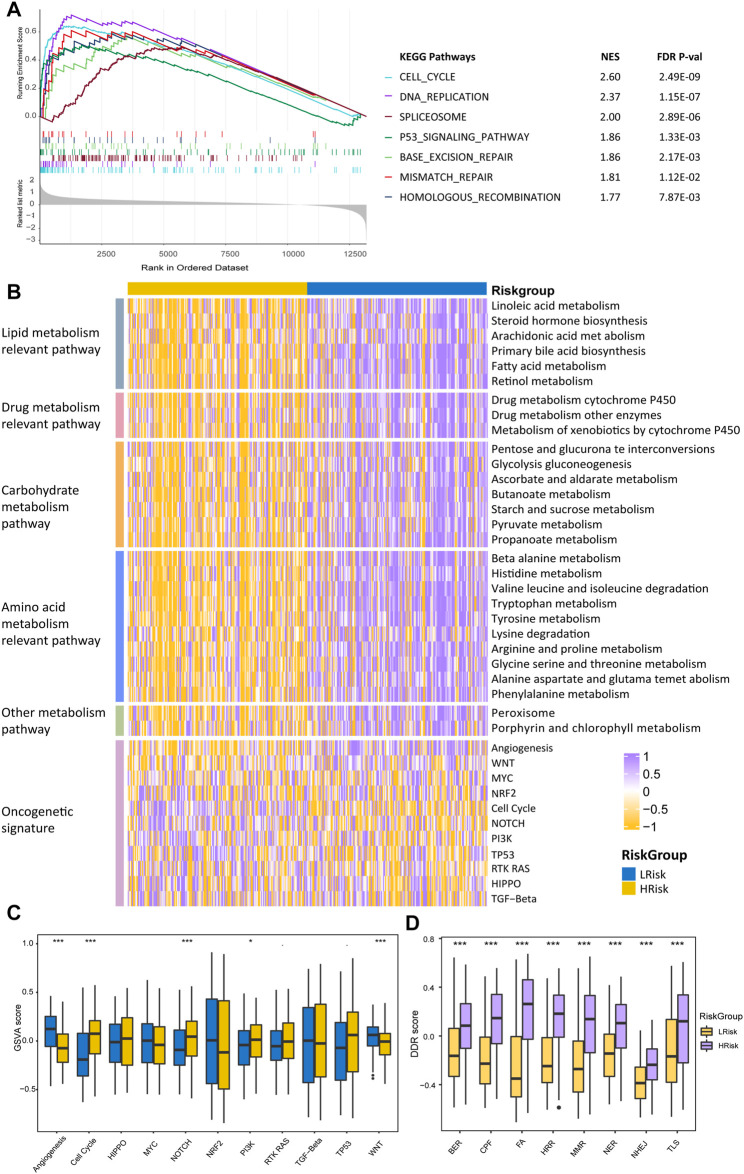
Differentially functional pathways between the HRisk and LRisk group in TCGA. **(A)** GSEA identified upregulated pathways in HRisk. **(B)** Heatmap of enrichment level calculated by GSVA for metabolism-related pathways derived from GSEA and oncogenic pathways. **(C,D)** Boxplot of oncogenic pathways **(C)** and DDR pathways **(D)** from GSVA of two riskgroups. Statistical *p* values were calculated by the Wilcoxon test and represented by. < 0.1, * <0.05, ** <0.01, and *** <0.001.

To better characterize the two HCC riskgroups, differential analyses were performed. Gene set enrichment analysis (GSEA) was conducted using the “clusterProfiler” package, and enrichment differences of pathways were significant if the FDR *p*-value < 0.15 and |NES| >1 in all four cohorts. The results indicated that 28 metabolism-relevant pathways were significantly upregulated in LRisk, while HRisk enriched in cell cycle, DNA replication, spliceosome, and DDR-relevant pathways (Figure 6A). Thus, the HRisk group presents upregulated cell cycle procession and DDR pathways which might contribute to the hyperproliferation and development of tumor cells. Pathway with significant differences in enrichment in all four cohorts was considered subclass specific pathway. GSVA was conducted to quantify and visualize the enrichment of 28 metabolism-related pathways which were classified into four specific metabolism signatures, including lipid metabolism relevant pathway, drug metabolism relevant pathway, carbohydrate metabolism relevant pathway, and amino acids metabolism relevant pathway (Figure 6A). Results confirmed that the LRisk group has significant upregulation of metabolism signatures, consistent with the results from GSEA.

To further investigate the activation of oncogenic pathways among HRisk and LRisk (Figure 6C), We found that the cell cycle oncogenic pathway was significantly activated in HRisk, while LRisk had a higher score of angiogenesis and Wnt activation-relevant pathways than HRisk (Figure 6D). Considering that the risk groups were divided based on DDR-relevant genes signature, we then decided to further explore whether different characteristics exist in distinct DDR pathways. Eight DDR core pathways were quantified using the GSVA algorithm. HRisk group exhibited higher expression for all eight pathways than LRisk. The same results were demonstrated in cohorts GSE14520, ICGC, and LIHC-CN (Supplementary Figures S5−S7).

### Relationship Between Riskscore and Somatic Mutation and Copy Number Variation

**FIGURE 7:**
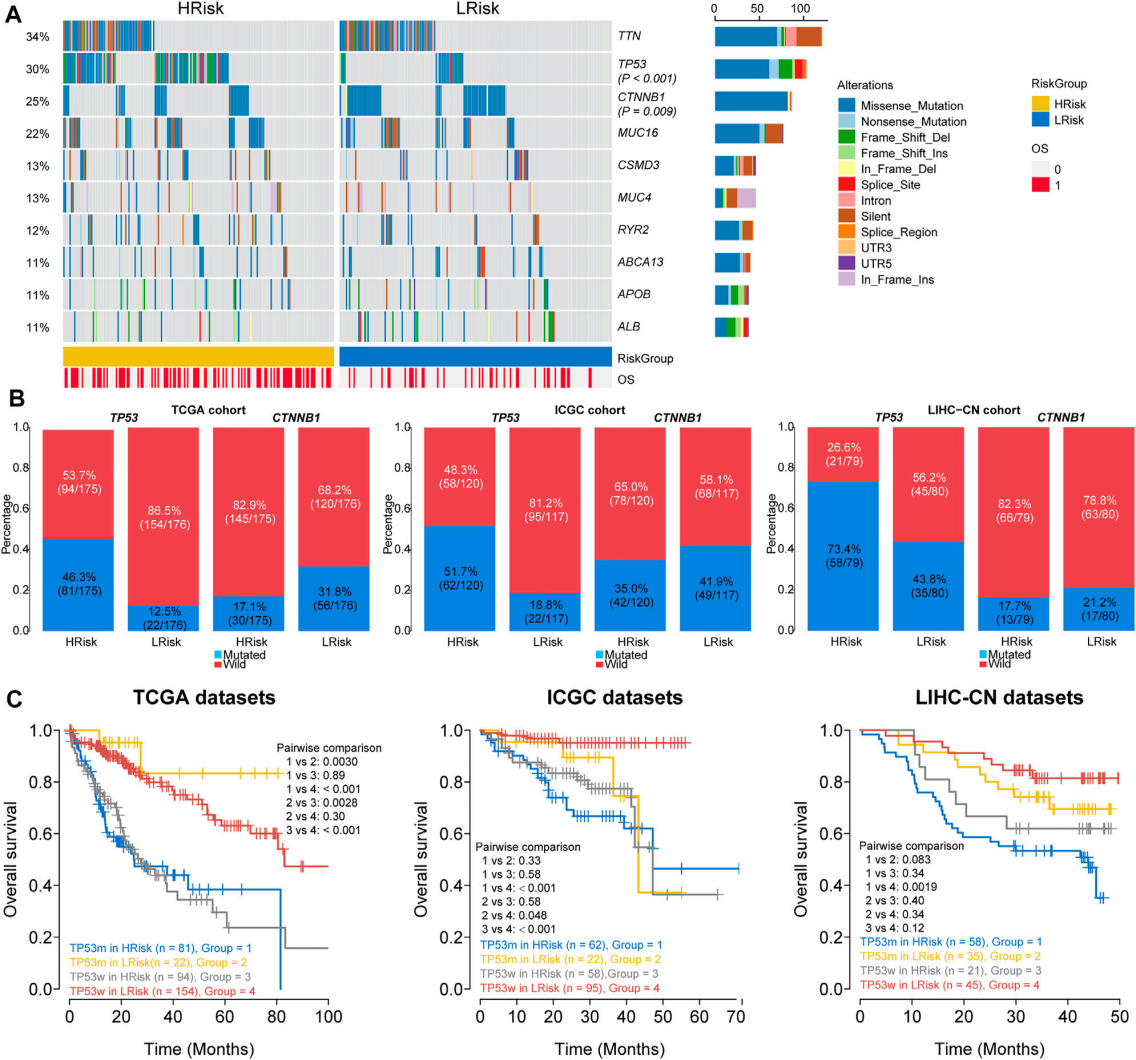
Analysis of mutation characteristics in HRisk and LRisk. **(A)** Oncoprint showing the mutational landscape of mutations with mutated greater than 10% in TCGA-HCC. Mutations of *TP53* and *CTNNB1* were significantly mutated in HRisk (Fisher’s exact test, *p* < 0.001) and LRisk (Fisher’s exact test, *p* = 0.009) groups, respectively. **(B)** Barplots showing the similar distribution of *TP53* and *CTNNB1* mutation between two riskgroups in TCGA cohort (*TP53*: 46.3 *vs*. 12.5%, *p* < 0.001; *CTNNB1*: 17.7 *vs*. 31.8%, *p* = 0.007), ICGC cohort (*TP53*: 51.7 *vs*. 18.8%, *p* < 0.001; *CTNNB1*:35.0 *vs*. 41.9%, *p* = 0.2883), and LIHC-CN cohort (*TP53*: 73.4 *vs*. 43.8%, *p* < 0.001; *CTNNB1*: 17.7 *vs*. 21.2%, *p* = 0.5439), respectively. **(C)** Kaplan–Meier curve analysis of overall survival is shown for patients classified according to *TP53* mutation status and the riskgroup in three cohorts. TP53w, *TP53*-sequence wild type; TP53m, *TP53*-sequence mutant type.

We finally investigated the genomic variations between two different risk groups in the TCGA cohort. To analyze whether differences exist in the somatic variations (10%) of HCC between two riskgroups, R package “maftools” was used (Figure 7A). The results showed that the HRisk group had significantly high *TP53* mutations and less *CTNNB1* mutations than the LRisk group (*TP53*: 46.3 *vs*. 12.5%, *p* < 0.001; *CTNNB1*: 17.7 *vs*. 31.8%, *p* = 0.007; Figure 7B), and we obtained consistent results in ICGC cohort (*TP53*: 51.7 *vs*. 18.8%, *p* < 0.001; *CTNNB1*:35.0 *vs*. 41.9%, *p* = 0.2883; Figure 7B) and LIHC-CN cohort (*TP53*: 73.4 *vs*. 43.8%, *p* < 0.001; *CTNNB1*: 17.7 *vs*. 21.2%, *p* = 0.5439; Figure 7B), respectively. Mutation in *TP53* is the most common genetic change in HCC, and patients with mutated *TP53* have shorter OS than those with wild-type *TP53*. Therefore, we conducted special subgroup analyses stratifying samples according to the combination of *TP53* mutation status and riskgroups. We found that some patients with mutant type *TP53* in the HRisk group had significantly shorter OS than those with mutant wild *TP53* in the HRisk group (*p* = 0.89 in TCGA, *p* = 0.58 in ICGC, *p* = 0.34 in LIHC-CN; Figure 7C), while patients with mutant type in the LRisk group had longer OS than those with wild type in the HRisk group (*p* = 0.0028 in TCGA; Figure 7C), but no significance could be calculated in ICGC and LIHC-CN datasets (*p* = 0.58 in ICGC, *p* = 0.40 in LIHC-CN; Figure 7C). These results confirm again that the prognosis model is robust and superior. Unfortunately, we could not find the mutation data of GSE14520.

**FIGURE 8:**
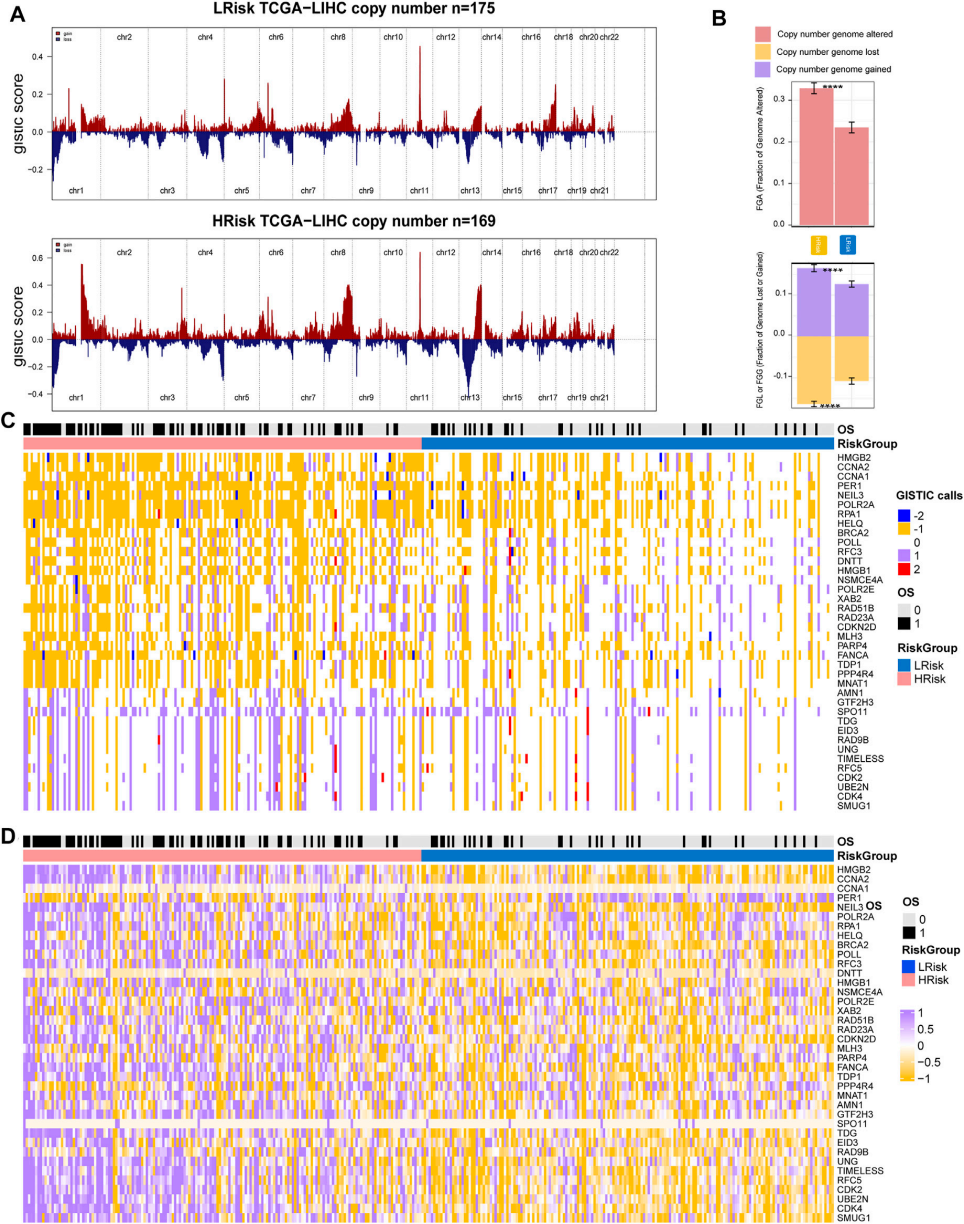
Integrative analysis of copy number alteration and gene expression profiling. **(A)** Copy number gains and deletions identified by GISTIC2.0 in HRisk and LRisk. **(B)** Distribution of fraction genome altered (FGA) and fraction genome loss/gain (FGG/FGL). Bar charts are presented as the mean ± standard error of the mean. **(C)** Heatmap of GISTIC calls of 38 DDR genes. **(D)** Heatmap showing the overexpression pattern of 38 DDR genes.

Copy number variations were a common form of genomic structural change, and an amount of research has demonstrated chromosomal abnormalities play key roles in HCC. GISTIC 2.0 was used to analyze the copy number of HRisk and LRisk in TCGA-HCC samples. We first calculate the FGA, FGL, and FGG scores to evaluate differences in chromosomal instability between the two risk groups. We found the LRisk group had significantly lower copy number loss or gain than HRisk, so it was obvious that LRisk had better chromosomal stability than HRisk (all, *p* < 0.001; Figure 8B). Next, we analyzed the copy number in different specific regions in LRisk and HRisk groups. The most frequent arm-level aberrations in the HRisk group identified were 13q, 11q, 4q, etc., for copy number loss, and significantly amplified regions in the HRisk were 1q, 8q, 3q, etc. (Figure 8A). Therefore, we decided to further explore the relationship between the copy number variations of specific DDR genes. The relationship between genes in eight core DDR pathways and their copy number alterations were calculated by Spearman analyses. The GISTIC calls of 38 DDR genes (R < −0.2 or R > 0.2) are shown in Figure 8C, and the corresponding expression of DDR genes are shown in Figure 8D. These results suggest that HRisk existed higher levels of copy number alterations, and both were associated with overexpression of DDR genes.

## Discussion

HCC remains a major public health concern in the world. Although continuous achievements in early detection, multimodal therapy, and surgery resection, the mortality is still high (Siegel et al., 2013). Additionally, an effective prognostic signature is very important for the prediction and individualized treatment of HCC. The DDR process is often exaggerated in HCC and affects the tumor development and therapeutic response of HCC patients. As a previous study, Li et al. has developed a seven-gene signature related to the DNA repair process to predict the prognosis of HCC (Li et al., 2019). Hence, it is of significance to construct a prediction model based on the expression profiles of DDR-related gene expression.

In this study, we built and validated a robust 23 DDR-related gene pair signature for HCC patients’ prognosis and precise treatment. The prognostic model was validated in the dependent TCGA, GSE14520, ICGC-JP, and LIHC-CN cohorts. We divided the HCC patients in each cohort into HRisk and LRisk according to the median cutoff of riskscore. Patients in the LRisk group had significantly longer OS than that in the HRisk group. The signature also demonstrated to be an independent risk factor for OS in HCC patients in four cohorts. Furthermore, subgroup analyses showed that the prognostic model could predict the outcomes of patients in different subgroups. The nomogram-integrated TNM stage and riskscore was established, which proved to be a better predictor than the TNM stage alone. These advantages could be helpful to make clinical decisions and make nomograms a superior tool for predicting prognosis.

Hepatitis B virus (HBV) is associated with the rapid progression of HCC, and its viral load has an adverse effect on overall survival (Yu and Kim, 2014). Studies have found that high HBV viral expression may stimulate the immune response in cervical cancer and favored the patient clinical outcome (Lu et al., 2019). In our study, we compressed the expression of four HBV oncoproteins into a comprehensive PCA-based score, *HBV*_*pca*_. 96 HBV-associated HCC patients in the TCGA cohort, while 43 patients in the LRisk group had a higher HBV viral load than those in the HRisk group. These results indicated that high HBV viral expression is significantly associated with a better prognosis, which is similar to the previous study. Then, we used the ssGSEA algorithm to analyze the immune infiltration between HRisk and LRisk. Although analysis of ssGSEA did not suggest significant greater levels of immune cell infiltrates in the LRisk group, we found that the LRisk group might be more beneficial from checkpoint blockade. The HBV genome can encode the four proteins, which include S, X, C, and P (Yang et al., 1995). There are extensive interactions between the HBV genome and the DDR pathway (Lee et al., 1995). Several findings suggest that HBV viral expression could disrupt the DNA repair pathways of infected hepatocytes (Ko and Ren, 2011; Ricardo-Lax et al., 2015; Schreiner and Nassal, 2017). For example, the hepatitis B virus X protein (HBx) is known to be a multifunctional protein encoded by HBV, playing a pivotal role in the development of viral-induced liver cancer (Becker et al., 1998). HBx might disturb several key cellular processes such as cell cycle, DNA repair, oxidative stress, transcription, protein degradation, signal transduction, and apoptosis. In some cases, components of the DDR network may be antiviral and have detrimental impacts on viral replication (Luftig, 2014; Weitzman and Fradet-Turcotte, 2018). In this study, HCC patients in the TCGA cohort were differentiated into two groups based on DDR-related gene expression profiles which showed different DDR patterns. HRisk group presented upregulated DDR-relevant pathway and cell cycle process, so we speculate DDR pathway might have interrupted the replication cycle of HBV proteins in HRisk so that the LRisk group have a higher HBV expression level was observed.

GSEA showed core DDR-related pathways (base excision repair, mismatch repair, homologous recombination), cell cycle, DNA replication, spliceosome, and p53 signaling pathway were distinctly enriched in HRisk. Patients in the DDR-activated subgroup were significantly related to the inferior prognosis. We also found several significant alterations of molecular characteristics between HRisk and LRisk groups. Common somatic changes include mutations p53 and beta-catenin are frequently detected repeatedly in HCC, resulting in activation of the Wnt signaling pathway and dysregulation of the cell cycle, respectively (Jacobs and Norton, 2021). *TP53* is the most frequently mutated in HCC, and patients with *TP53* mutations had a poorer prognosis compared with patients with wild-type *TP53* (Villanueva and Hoshida, 2011). We observed that the *TP53* gene was more frequently mutated in the HRisk. In contrast to the *TP53* gene, a larger proportion of LRisk carried *CTNNB1* mutations. *CTNNB1* mutations in HCC were mutually exclusive with *TP53* (Calderaro et al., 2017), and mutation-induced activation of *CTNNB1* expression is the dominant cause of Wnt activation (Takagi et al., 2008). In addition, LRisk had a significantly higher score of Wnt activation-relevant signature, which may be activated by mutated *CTNNB1*. LRisk group was significantly involved in many metabolism pathways, including lipid metabolism, drug metabolism, carbohydrate metabolism, and amino acid metabolism relevant pathways, indicating that patients in the LRisk group hold a normal metabolic process (e.g., fatty acid, gluconeogenesis, and histidine) of liver and the activation of metabolism relevant signatures is associated with a favorable prognosis in patients. These findings are in keeping with a previous proteogenomics study, which proved that *CTNNB1*-mutated tumor was concentrated with various metabolic processes, including amino acid metabolism, glycolysis/gluconeogenesis, and drug metabolism (Gao et al., 2019). Along with mutations, chromosomal abnormalities are frequent genetic events in HCC (Schulze et al., 2016). In particular, broad genomic deletions have been noted for 1p, 4q, 6q, 8p, 13q, 16p, and 17q and gains for 1q, 6p, 8q, 17q, and 20q (Jacobs and Norton, 2021). We found that HRisk had significantly higher copy number alterations than LRisk, suggesting that HRisk existed a deeper degree of chromosomal instability. Somatic copy alterations (SCNAs) are widespread in human cancers that promote tumor initiation and progression (Beroukhim et al., 2010). Higher levels of SCNAs are associated with an increased expression level of the cell cycle (Davoli et al., 2017). Our results indicated that the HRisk group with a significantly higher cell proliferation signature is related to higher SCNA levels of DDR genes and expression. Altogether, our study provides a comprehensive overview of molecular characteristics between HRisk and LRisk.

Although there are several therapeutic options for HCC, chemotherapy is one of the most important treatment modalities for advanced HCC. However, the efficacy of chemotherapy remains unsatisfactory, so it is necessary to identify a signature to better predict chemotherapy responses of HCC patients. Interestingly, the two groups had different sensitivity to common chemotherapy for treating HCC. Potential drugs for HRisk and LRisk patients were then investigated. Cyclin-dependent kinases provided by a family of serine kinases primarily control the eukaryotic cell cycle (Aprelikova et al., 1995). Both CDK9_5038 and CDK9_5576 are CDK9 inhibitors. Bleomycin is classified as an “antitumor antibiotic” drug, and the drug works by binding to DNA that could generate lesions on both strands of DNA (Chen and Stubbe, 2005). The epidermal growth factor receptor (EGFR) plays a central role in the development and progression of different cancers. Afatinib and gefitinib are the currently available EGFR-tyrosine kinase inhibitors (EGFR-TKIs), which have been approved so far for non-small cell lung patients. In this study, patients in HRisk may be more sensitive to CDK9 inhibitors and Bleomycin, while patients in LRisk may be more sensitive to EGFR-TKIs, which should be validated in future clinical trials.

There are several limitations to our study. First, although our research was validated by other independent cohorts, they were all retrospective data. Second, highly heterogeneous, intratumoral heterogeneity in HCC might have an impact on the DRGP riskscore in each tumor, so its significance for clinical translation therapy needs to be further confirmed. Moreover, we determined several drugs that have different sensitivity in HCC patients. However, investigations about the antitumor effects of some drugs are lacking.

## Conclusion

A signature based on the 23 DDR-related gene pairs was successfully constructed, which stratifies HCC patients into two riskgroups with different survival outcomes. Enrichment analysis, CNV, gene mutation, and tumor immune environmental analyses were conducted between HRisk and LRisk. The prediction of therapy sensitivity may be helpful to clinicians in selecting patients that could benefit from further treatments. These findings may provide a novel prognostic signature for HCC from a DDR perspective and enhance biological understanding and clinical strategies in HCC.

## Data Availability

The datasets presented in this study can be found in online repositories. The names of the repository/repositories and accession number(s) can be found in the article/Supplementary Material.
