# Supplementary material for: Identification of a DNA Damage Response and Repair-Related Gene-Pair Signature for Prognosis Stratification Analysis in Hepatocellular Carcinoma
Source: Front Pharmacol. 2022 Apr 5;13:857060. doi: 10.3389/fphar.2022.857060 (PMC9038539; doi:10.3389/fphar.2022.857060)
Supplement: Supplementary file 1 [file DataSheet1.docx]

**Identification of a DNA damage** **response and repair related gene-pair signature and prognosis stratification analysis in hepatocellular carcinoma**

**Supplementary Figures and Tables**

**Supplementary Tables S1-3……………………………………………Page 2**

**Supplementary Figures S1-7 …………………………………………. Page 5**

**SUPPLEMENTARY TABLES**

**Supplementary Table S1. 384 DDR gene list.**

| *AATF* | *BARD1* | *CCND3* | *CHEK2* | *CUL5* | *E2F1* | *FANCF* | *HERC2* | *RBX1* | *SMARCA2* |
| --- | --- | --- | --- | --- | --- | --- | --- | --- | --- |
| *ACD* | *BAX* | *CCNH* | *CHRNA4* | *CYP1A1* | *E2F2* | *FANCG* | *HES1* | *RECQL* | *SMARCA4* |
| *ACTL6A* | *BAZ1A* | *CDC14B* | *CIB1* | *DAPK1* | *E2F4* | *FANCL* | *HIC1* | *RECQL4* | *SMARCA5* |
| *ACTR8* | *BAZ1B* | *CDC25A* | *CINP* | *DAXX* | *E2F6* | *FEN1* | *HIST3H2A* | *RECQL5* | *SMARCB1* |
| *AKT1* | *BCAS2* | *CDC25B* | *CLK2* | *DBF4* | *EGFR* | *FHIT* | *HLTF* | *RELA* | *SMARCC1* |
| *ALKBH1* | *BCCIP* | *CDC25C* | *COPS2* | *DCLRE1A* | *EP300* | *FOS* | *HMGB1* | *REV1* | *SMARCC2* |
| *ANKRD28* | *BLM* | *CDC5L* | *COPS3* | *DCLRE1B* | *ERBB2* | *FOXM1* | *HMGB2* | *REV3L* | *SMARCD1* |
| *APEX1* | *BRAP* | *CDC6* | *COPS4* | *DCLRE1C* | *ERCC1* | *FTO* | *HUS1* | *RFC1* | *SMARCE1* |
| *APEX2* | *BRCA1* | *CDH13* | *COPS6* | *DDB1* | *ERCC2* | *FZR1* | *HUWE1* | *RFC2* | *SMC1A* |
| *APTX* | *BRCA2* | *CDK7* | *COPS7A* | *DDB2* | *ERCC3* | *GADD45A* | *IDH1* | *RFC3* | *SMC2* |
| *ARID1A* | *BRCC3* | *CDKN1B* | *COPS7B* | *DDR1* | *ERCC4* | *GADD45G* | *IFI16* | *RFC4* | *SMC3* |
| *ASCC3* | *BRD7* | *CEBPG* | *COPS8* | *DDX1* | *ERCC5* | *GSTP1* | *IGF1* | *RFC5* | *SMC4* |
| *ASF1A* | *BRIP1* | *CEP164* | *CREB1* | *DEK* | *ERCC6* | *GTF2E2* | *IGHMBP2* | *RFWD3* | *SMC5* |
| *ASTE1* | *BTG2* | *CEP170* | *CREBBP* | *DHX9* | *ERCC8* | *GTF2H1* | *IKBKG* | *RIF1* | *SMC6* |
| *ATF2* | *BUB1* | *CETN2* | *CRY1* | *DKC1* | *ESR1* | *GTF2H3* | *INTS3* | *RMI1* | *SMG6* |
| *ATM* | *BUB1B* | *CHAF1A* | *CRY2* | *DOT1L* | *ETS1* | *GTF2H4* | *IRS1* | *RNF4* | *SMUG1* |
| *ATR* | *CASP3* | *CHAF1B* | *CSNK1D* | *DTL* | *EXO1* | *GTF2H5* | *JUN* | *RNF8* | *SOX4* |
| *ATRX* | *CCNB2* | *CHD1L* | *CSNK1E* | *DUSP3* | *FANCA* | *H2AFX* | *KIN* | *RNMT* | *STAG1* |
| *ATXN3* | *CCNC* | *CHD4* | *CUL3* | *DUT* | *FANCC* | *HDAC1* | *LIG1* | *RPA1* | *STAG2* |
| *BAP1* | *CCND2* | *CHEK1* | *CUL4A* | *DYRK2* | *FANCE* | *HDAC2* | *LIG3* | *RPA2* | *SUMO1* |
| *LIG4* | *MSH2* | *NCAPH* | *NSMCE4A* | *PER1* | *POLE3* | *PPP2R5D* | *RAD50* | *RPA3* | *SUMO2* |
| *MBD4* | *MSH3* | *NCAPH2* | *NTHL1* | *PLK1* | *POLG* | *PPP2R5E* | *RAD51* | *RPS27A* | *SUMO3* |
| *MC1R* | *MSH5* | *NCOA6* | *NUDT1* | *PLK3* | *POLG2* | *PPP4C* | *RAD51AP1* | *RPS27L* | *TCEA1* |
| *MCPH1* | *MSH6* | *NEIL1* | *NUDT15* | *PMS1* | *POLH* | *PPP4R1* | *RAD51C* | *RRM1* | *TDG* |
| *MCRS1* | *MTA1* | *NEIL3* | *NUDT18* | *PMS2* | *POLI* | *PPP4R2* | *RAD52* | *RRM2* | *TDP1* |
| *MDC1* | *MUM1* | *NEK1* | *OGG1* | *PNKP* | *POLL* | *PPP6C* | *RAD54B* | *RUVBL1* | *TELO2* |
| *MEN1* | *MUS81* | *NEK11* | *OTUB1* | *POLA1* | *POLM* | *PRKDC* | *RAD54L* | *RUVBL2* | *TEP1* |
| *MGMT* | *MUTYH* | *NFATC2IP* | *PALB2* | *POLB* | *POLQ* | *PRPF19* | *RAD54L2* | *SETD2* | *TERF1* |
| *MLH1* | *MVP* | *NFKB1* | *PARK7* | *POLD1* | *POT1* | *PSMD3* | *RAD9A* | *SETMAR* | *TERF2* |
| *MLH3* | *MYC* | *NFRKB* | *PARP1* | *POLD2* | *PPM1D* | *PTEN* | *RASSF1* | *SETX* | *TERF2IP* |
| *MNAT1* | *NBN* | *NHEJ1* | *PARP2* | *POLD3* | *PPP1CA* | *PTTG1* | *RB1* | *SFPQ* | *TERT* |
| *MORF4L1* | *NCAPD2* | *NME1* | *PARP3* | *POLD4* | *PPP2R2A* | *RAD1* | *RBBP4* | *SHPRH* | *TFPT* |
| *MORF4L2* | *NCAPD3* | *NONO* | *PARP4* | *POLDIP3* | *PPP2R5A* | *RAD17* | *RBBP7* | *SIRT1* | *TINF2* |
| *MPG* | *NCAPG* | *NPM1* | *PAXIP1* | *POLE* | *PPP2R5B* | *RAD23A* | *RBBP8* | *SIRT6* | *TNKS* |
| *MRPL40* | *NCAPG2* | *NSMCE2* | *PCNA* | *POLE2* | *PPP2R5C* | *RAD23B* | *RBM14* | *SLC30A9* | *TOP1* |
| *TOP2A* | *TP53* | *TYMS* | *UBE2A* | *UBE2V2* | *WDR48* | *XPA* | *XRCC3* | *YWHAB* | *XRCC2* |
| *TOP2B* | *TP53BP1* | *UBB* | *UBE2B* | *UIMC1* | *WEE1* | *XPC* | *XRCC4* | *YWHAE* | *XAB2* |
| *TOP3A* | *TP73* | *UBC* | *UBE2I* | *UNG* | *WRN* | *XRCC1* | *XRCC5* | *XRCC6* | *USP1* |
| *TOPBP1* | *TTK* | *UBD* | *UBE2N* |  |  |  |  |  |  |

| **Supplementary Table S2. Demographic and clinic characteristic descriptions for HCC patients in different datasets.** | | | | | | |
| --- | --- | --- | --- | --- | --- | --- |
| **Characteristics^a^** | **TCGA-training** | **TCGA-testing** | **TCGA** | **GEO14520** | **ICGC** | **LIHC-CN** |
| Number of samples | 246 | 105 | 351 | 219 | 240 | 159 |
| Number of Death (%) | 85(34.6) | 37(35.2) | 122(34.8) | 84(38.4) | 43(17.9) | 56(35.2) |
| Age (Years)^b^ | 60.1±14.1 | 59.0±13.0 | 59.3±13.4 | 50.7±10.6 | 67.5±10.1 | 54.0±10.9 |
| Gender |  |  |  |  |  |  |
| Female | 83 | 33 | 116 | 30 | 61 | 31 |
| Male | 163 | 72 | 235 | 189 | 179 | 128 |
| Grade |  |  |  |  |  |  |
| G1 | 40 | 12 | 52 | NA | NA | NA |
| G2 | 113 | 57 | 170 | NA | NA | NA |
| G3 | 83 | 29 | 112 | NA | NA | NA |
| G4 | 9 | 3 | 12 | NA | NA | NA |
| TNM Stage |  |  |  |  |  |  |
| I | 118 | 48 | 166 | 93 | 31 | 91 |
| II | 54 | 26 | 80 | 77 | 165 | 14 |
| III | 57 | 23 | 80 | 49 | 23 | 52 |
| IV | 3 | 1 | 4 | NA | 1 | 2 |
| BCLC Stage |  |  |  |  |  |  |
| 0/A | NA | NA | NA | 168 | NA | 68 |
| B/C | NA | NA | NA | 51 | NA | 91 |
| ^a^ Sum of frequency numbers may not equal to the total sample size due to missing or unpredictable values | | | | | | |
| ^b^ Age is represented as mean ± standard deviation | | | | | | |

**Supplementary Table S3. Summary table for LASSO Cox regression analyses.**

| **DRGP1** | **DRGP2** | **Coef** | **HR** | **lower** | **upper** | **FDR-*P*** |
| --- | --- | --- | --- | --- | --- | --- |
| *FOXM1* | *PER1* | -0.1795 | 0.29 | 0.19 | 0.42 | 7.29E-07 |
| *NCAPG* | *RFC3* | -0.2559 | 0.39 | 0.27 | 0.56 | 2.78E-04 |
| *CDC6* | *MDC1* | -0.3012 | 0.41 | 0.27 | 0.61 | 1.58E-03 |
| *RECQL* | *TP53* | -0.1388 | 0.44 | 0.28 | 0.68 | 8.57E-03 |
| *DBF4* | *POLG2* | -0.4252 | 0.45 | 0.3 | 0.68 | 4.34E-03 |
| *GTF2H3* | *SIRT1* | -0.2995 | 0.46 | 0.28 | 0.76 | 2.73E-02 |
| *POLH* | *TP53* | -0.3232 | 0.5 | 0.33 | 0.78 | 2.44E-02 |
| *MSH2* | *POLH* | -0.5176 | 0.52 | 0.33 | 0.8 | 3.24E-02 |
| *GTF2E2* | *PER1* | -0.2696 | 0.53 | 0.37 | 0.75 | 1.08E-02 |
| *FANCL* | *NUDT15* | -0.2874 | 0.54 | 0.36 | 0.8 | 2.59E-02 |
| *E2F6* | *PPM1D* | -0.0144 | 0.58 | 0.4 | 0.83 | 3.15E-02 |
| *RBBP4* | *RPA1* | -0.101 | 0.59 | 0.41 | 0.85 | 4.05E-02 |
| *CRY1* | *MUTYH* | 0.2466 | 1.78 | 1.24 | 2.55 | 2.30E-02 |
| *CCNH* | *PPP2R5E* | 0.1717 | 1.8 | 1.23 | 2.63 | 2.85E-02 |
| *AKT1* | *SOX4* | 0.128 | 1.88 | 1.3 | 2.74 | 1.61E-02 |
| *RFC1* | *SOX4* | 0.0335 | 1.91 | 1.29 | 2.81 | 1.83E-02 |
| *MSH3* | *MUS81* | 0.2761 | 1.95 | 1.26 | 3.04 | 3.09E-02 |
| *POLE2* | *TTK* | 0.1369 | 2.18 | 1.48 | 3.2 | 3.59E-03 |
| *ERCC4* | *NEK11* | 0.2385 | 2.19 | 1.4 | 3.41 | 1.28E-02 |
| *CREBBP* | *SMC4* | 0.1114 | 2.33 | 1.53 | 3.56 | 4.23E-03 |
| *MGMT* | *YWHAB* | 0.399 | 2.4 | 1.67 | 3.45 | 6.68E-04 |
| *GSTP1* | *RECQL4* | 0.4197 | 2.44 | 1.6 | 3.71 | 2.32E-03 |
| *MGMT* | *PRKDC* | 0.1417 | 3.55 | 2.26 | 5.57 | 6.71E-05 |

**SUPPLEMENTARY FIGURES**

**
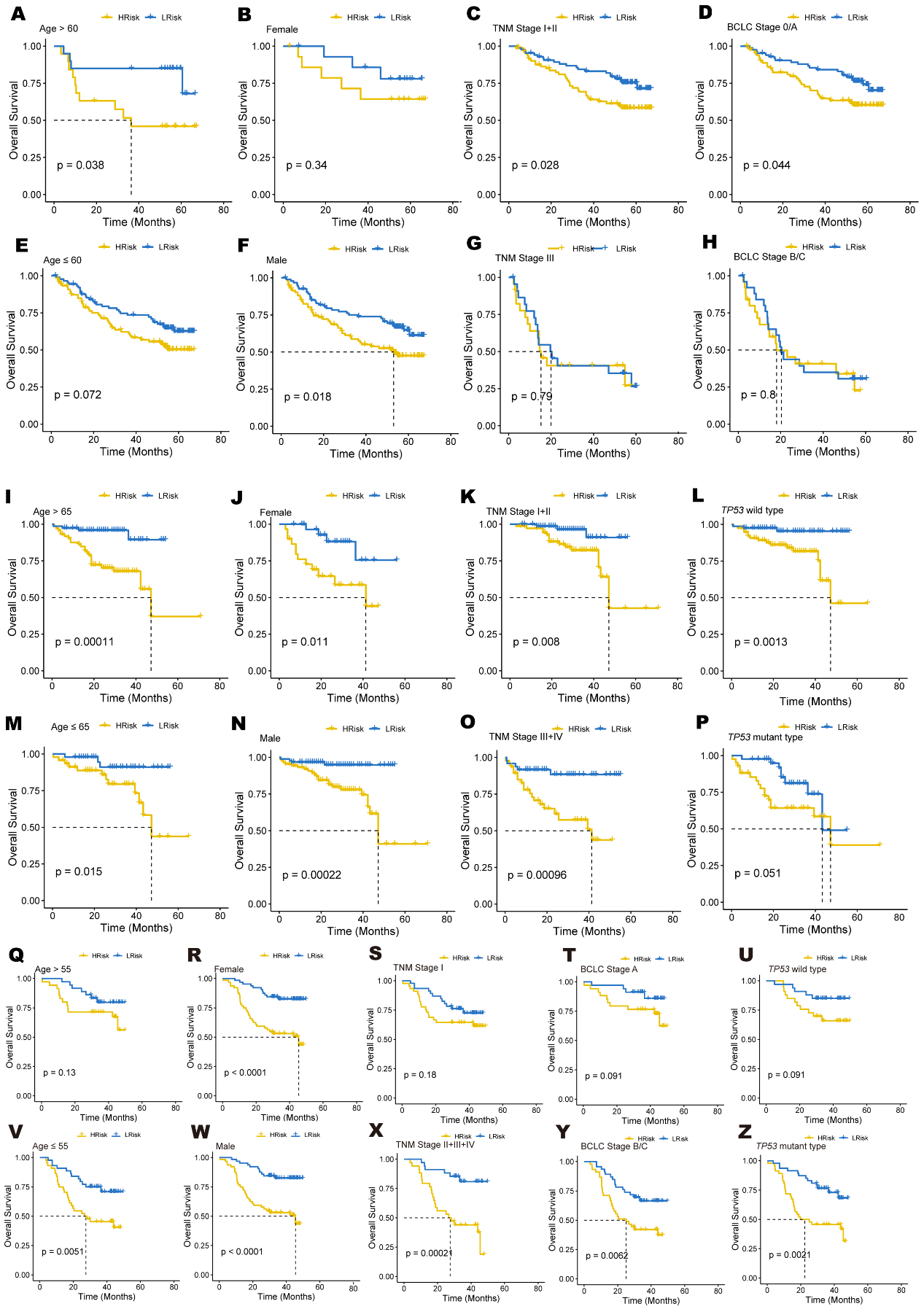
**

**Supplementary Figure S1.** Kaplan–Meier curves analyses of different clinical subgroups in GSE14520, ICGC, LIHC-CN cohorts. **(A-H)** Patients in GSE14520 datasets were classified into (A) Age > 60 years, (B) Gender: Female, (C) TNM Stage: I+II, (D) BCLC Stage: 0/A, (E) Age ≤ 60 years, (F) Gender: Male, (G) TNM Stage: III, (H) BCLC Stage: B/C. **(I-P)** Patients in ICGC datasets were classified into (I) Age > 65 years, (J) Gender: Female, (K)TNM Stage: I+II, (L) *TP53* wild type, (M) Age ≤ 65 years, (N) Gender: Male, (O) TNM Stage: III+IV, (P) *TP53* mutant type. **(Q-Z)** Patients in LIHC-CN datasets were classified into (Q) Age > 55 years, (R) Gender: Female, (S) TNM Stage: I, (T) BCLC Stage: 0/A, (U) *TP53* wild type, (V) Age ≤ 55 years, (W) Gender: Male, (X) TNM Stage: II+III+IV, (Y) BCLC Stage: B/C, (Z) *TP53* mutant type.


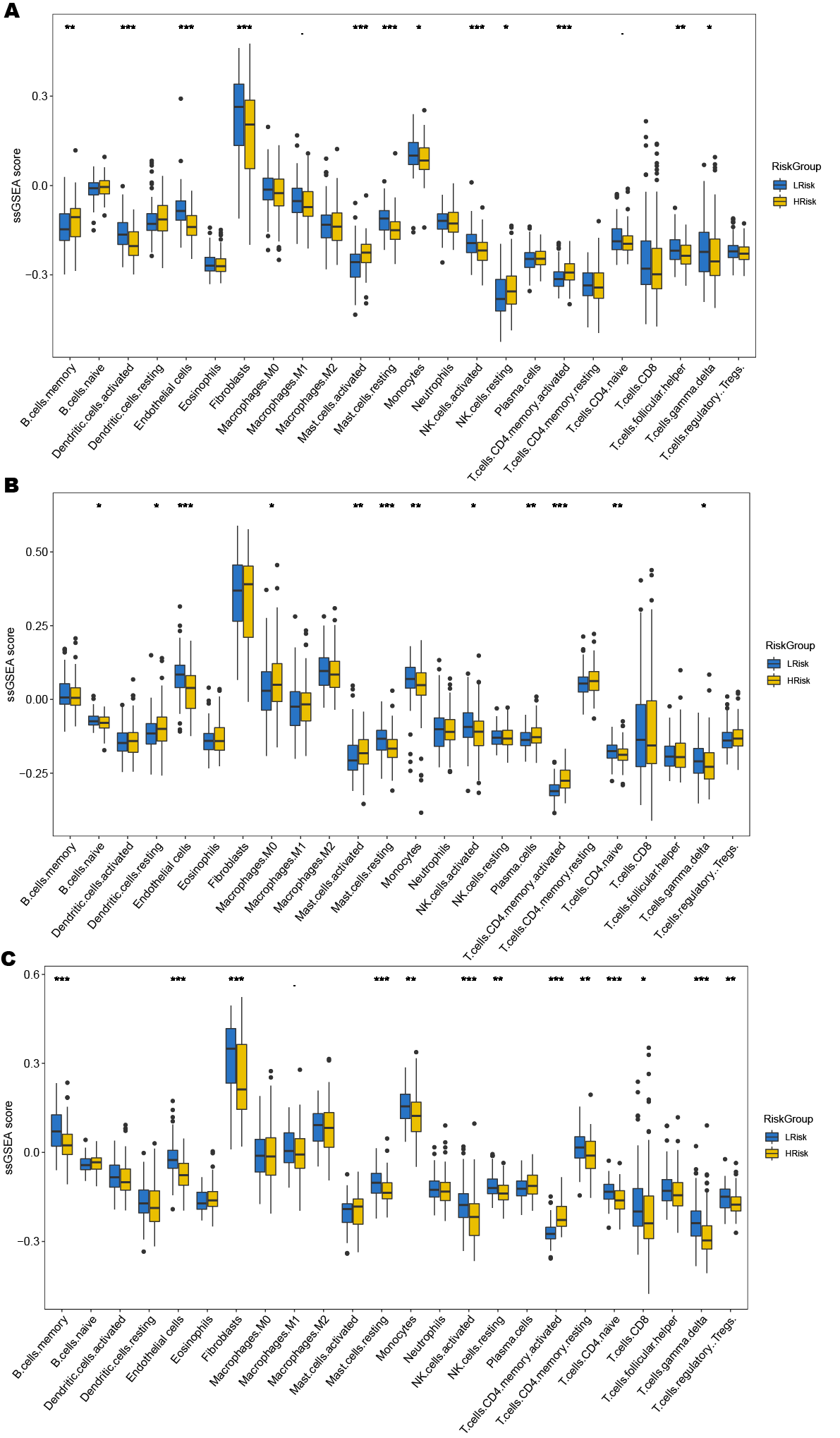


**Supplementary Figure S2.** Immune infiltration between HRisk and Lrisk group in GSE14520 **(A)**, ICGC **(B)**, LIHC-CN **(C)** cohorts. Boxplot for TCGA cohort showing the enrichment level of 24 microenvironment cell types between HRisk (yellow) and LRisk (Blue) groups. Statistical P values were calculated by wilcoxon test and represented by . < 0.1, * < 0.05, ** < 0.01 and *** < 0.001.


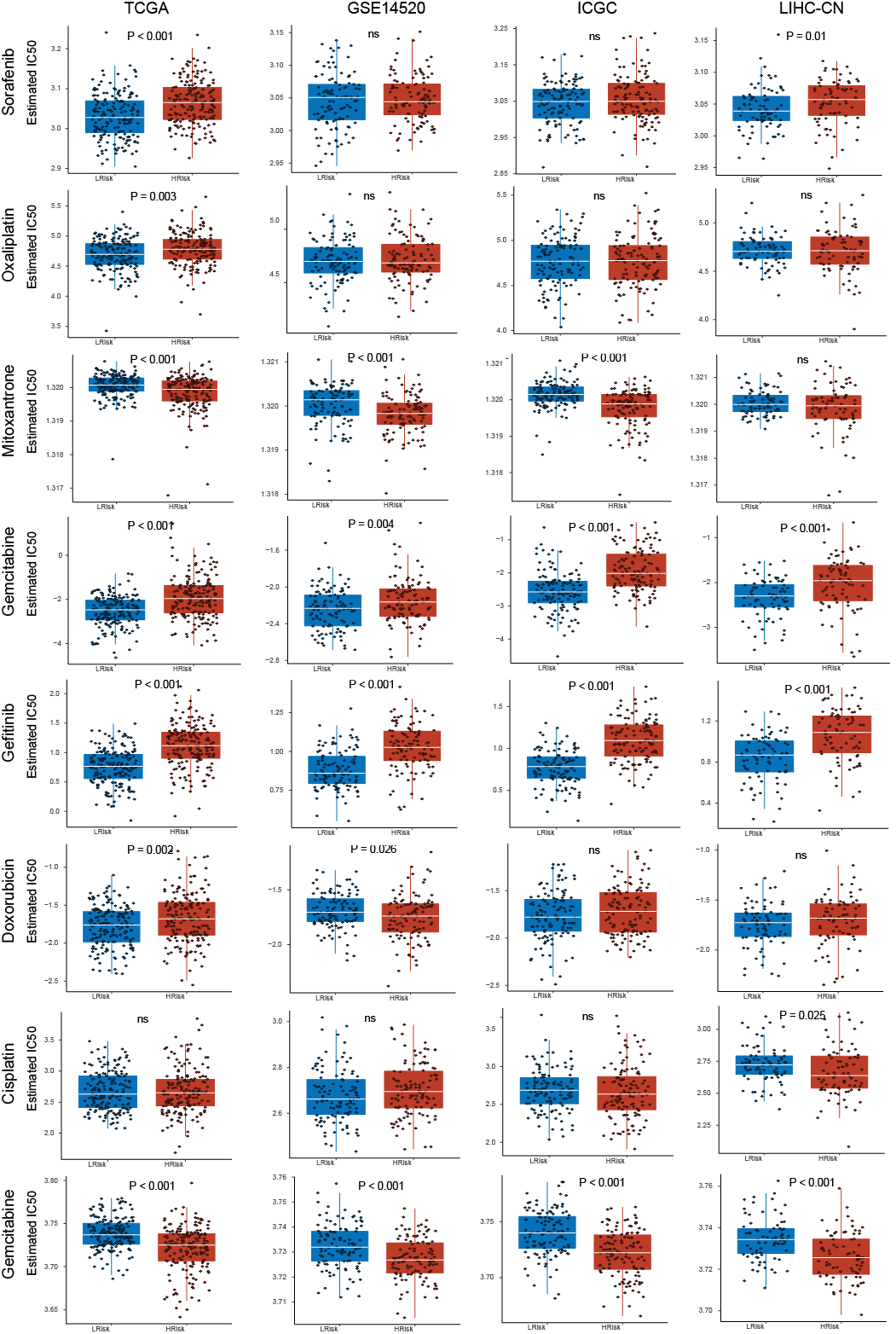


**Supplementary Figure S3.** Differential chemotherapeutic response in LRisk and HRisk. The box plots of the estimated IC_50_ of HRisk (red box) and LRisk (blue box) group for eight common drugs used in HCC are shown, (including Cisplatin, 5-Fluorouracil, Gemcitabine, Oxaliplatin, Doxorubicin, Mitoxantrone, Gefitinib and Sorafenib. Statistical *P* values are calculated by Wilcoxon test.


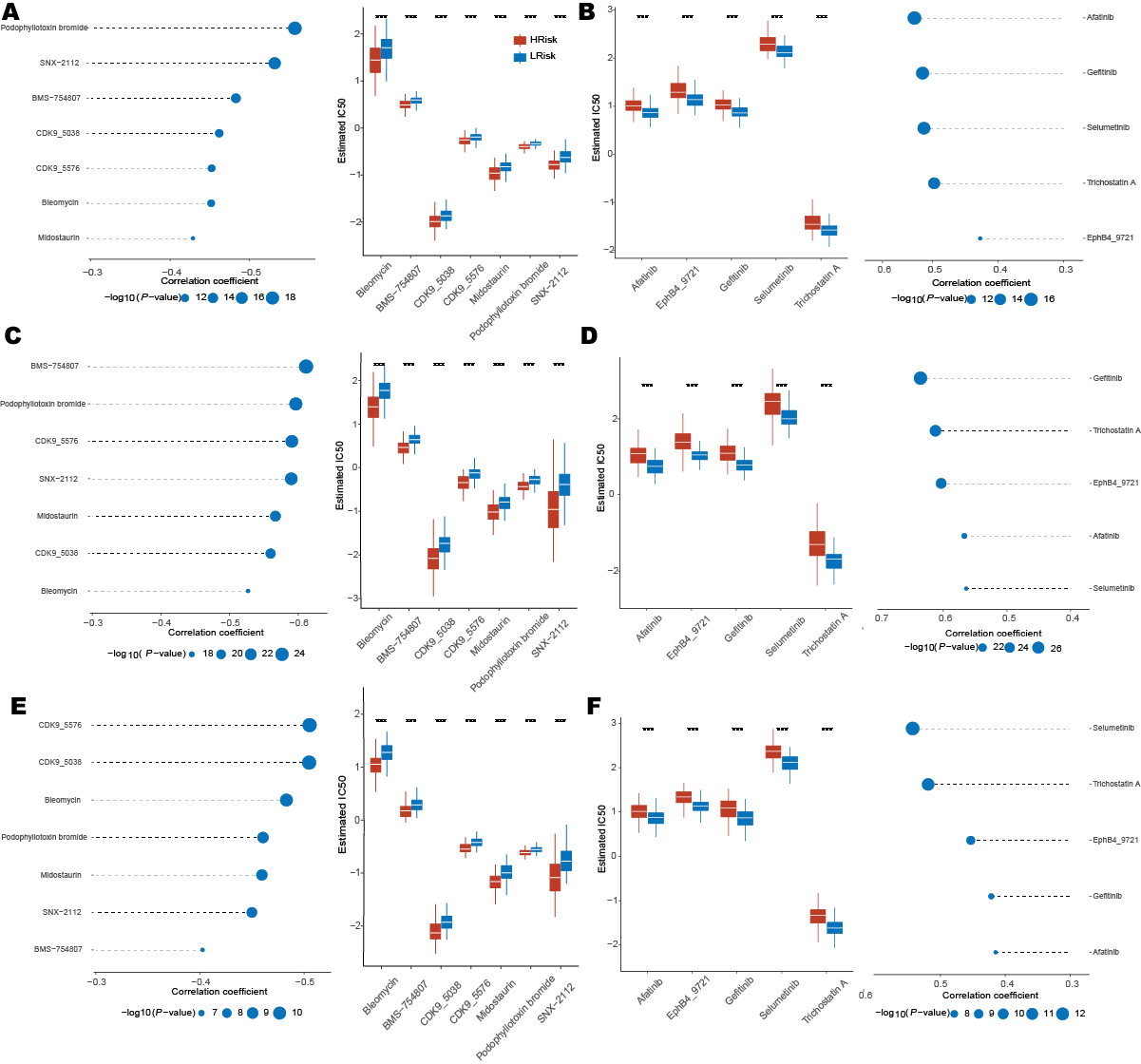


**Supplementary Figure S4.** Identification of candidate compounds with higher drug sensitivity in GSE14520, ICGC, LIHC-CN patients of HRisk and LRisk groups. The results of Spearman’s correlation analysis and boxplots for the distribution of seven HRisk sensitivity drugs response analysis in GSE14520 **(A)**, ICGC **(C)** LIHC-CN **(E)** cohorts. The results of Spearman’s correlation analysis and boxplots for the distribution of five LRisk sensitivity drugs response analysis in GSE14520 **(B)**, ICGC **(D)** LIHC-CN **(F)** cohorts.


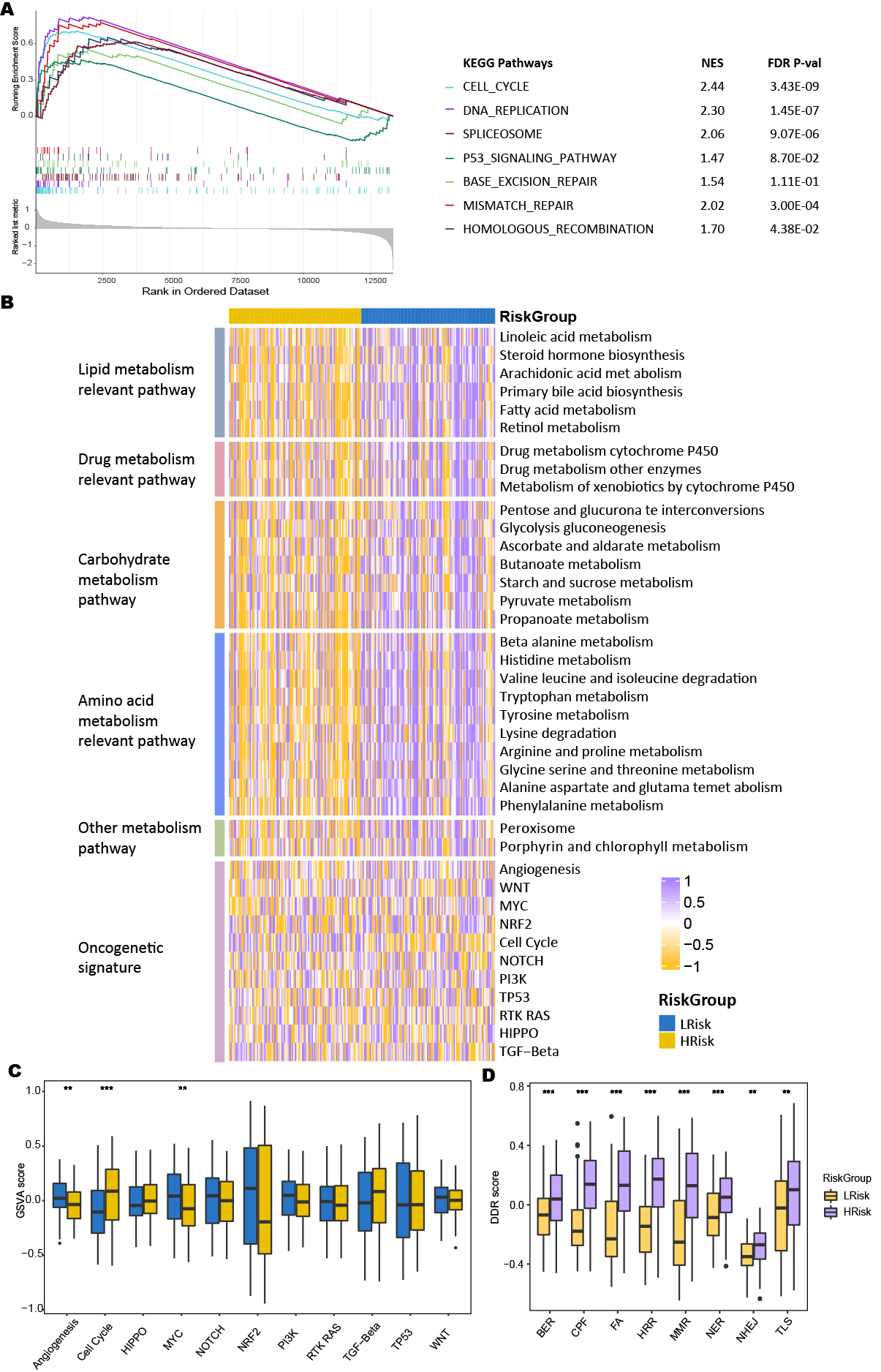


**Supplementary Figure S5.** Differentially functional pathways between the HRisk and LRisk in GSE14520. **(A)** GSEA identified upregulated pathways in HRisk. **(B)** Heatmap of enrichment level calculated by gene set variation analysis (GSVA) for metabolism-related pathways derived from GSEA and oncogenic pathways. **(C-D)** Boxplot of oncogenic pathways (C) and DDR pathways (D) from GSVA of two riskgroups. Statistical *P* values were calculated by wilcoxon test and represented by . < 0.1, * < 0.05, ** < 0.01 and *** < 0.001.


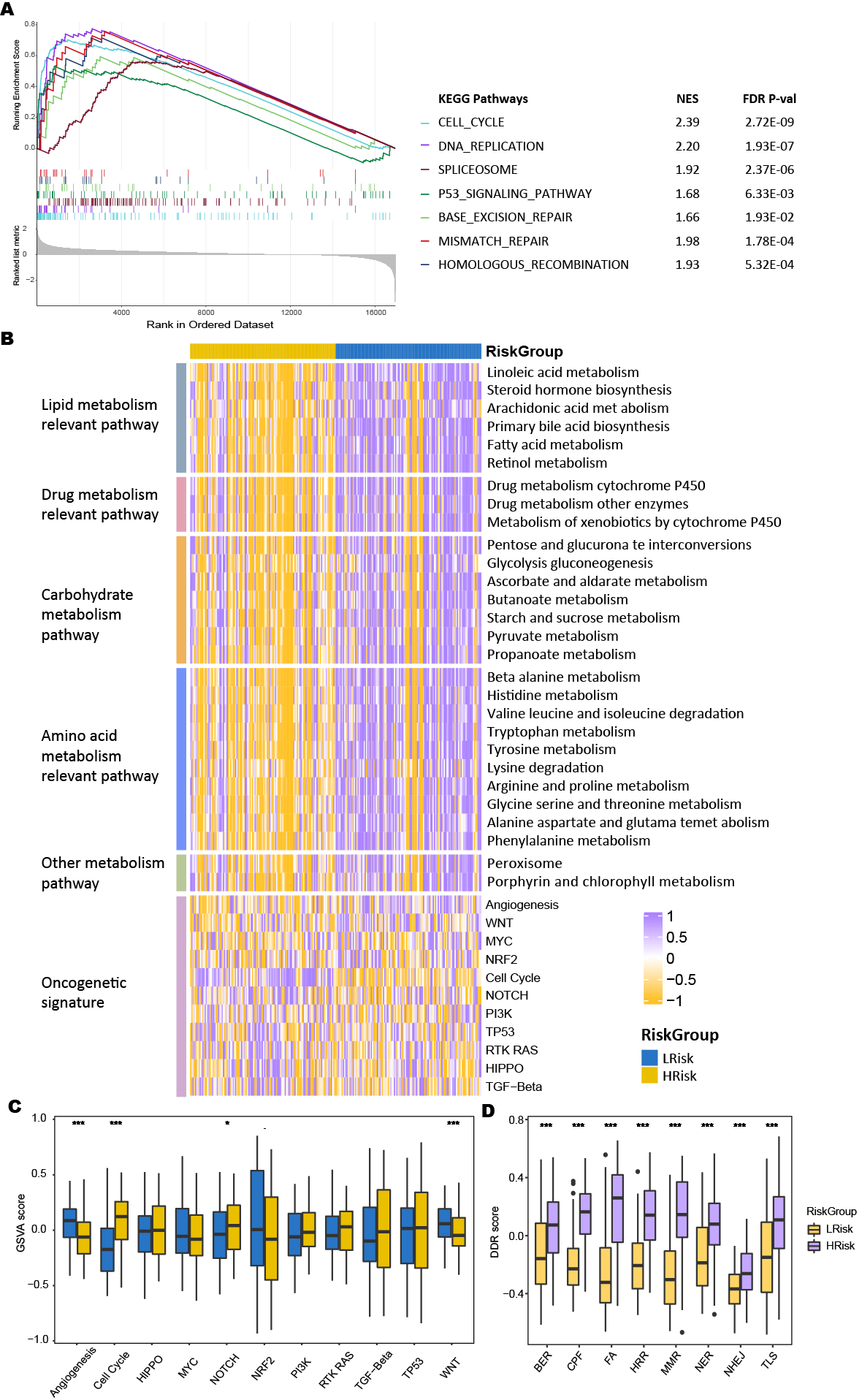


**Supplementary Figure S6.** Differentially functional pathways between the HRisk and LRisk in ICGC cohort. **(A)** GSEA identified upregulated pathways in HRisk. **(B)** Heatmap of enrichment level calculated by gene set variation analysis (GSVA) for metabolism-related pathways derived from GSEA and oncogenic pathways. **(C-D)** Boxplot of oncogenic pathways (C) and DDR pathways (D) from GSVA of two riskgroups. Statistical *P* values were calculated by wilcoxon test and represented by . < 0.1, * < 0.05, ** < 0.01 and *** < 0.001.


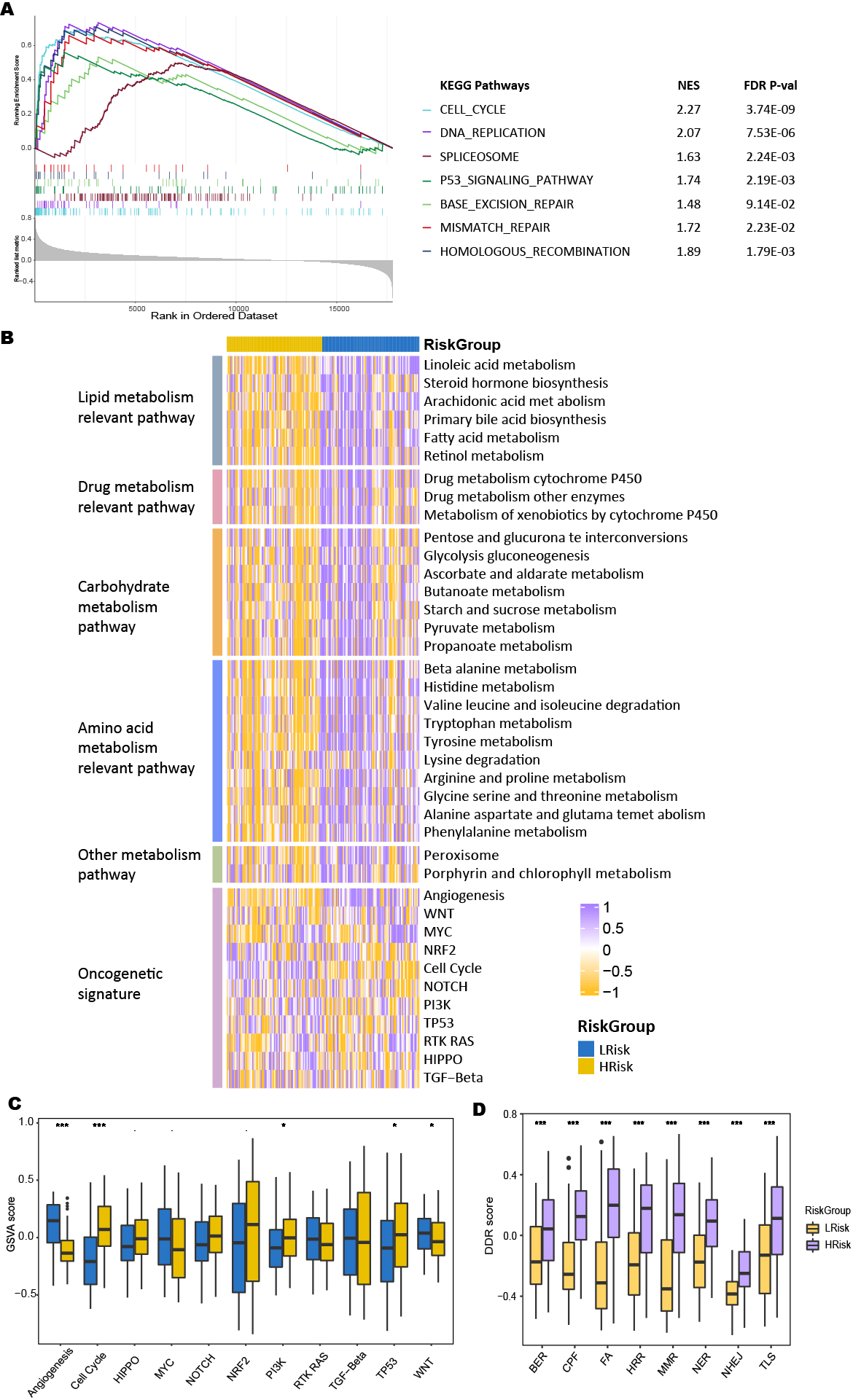


**Supplementary Figure S7.** Differentially functional pathways between the HRisk and LRisk in LIHC-CN cohort. **(A)** GSEA identified upregulated pathways in HRisk. **(B)** Heatmap of enrichment level calculated by gene set variation analysis (GSVA) for metabolism-related pathways derived from GSEA and oncogenic pathways. **(C-D)** Boxplot of oncogenic pathways (C) and DDR pathways (D) from GSVA of two riskgroups. Statistical *P* values were calculated by wilcoxon test and represented by . < 0.1, * < 0.05, ** < 0.01 and *** < 0.001.
